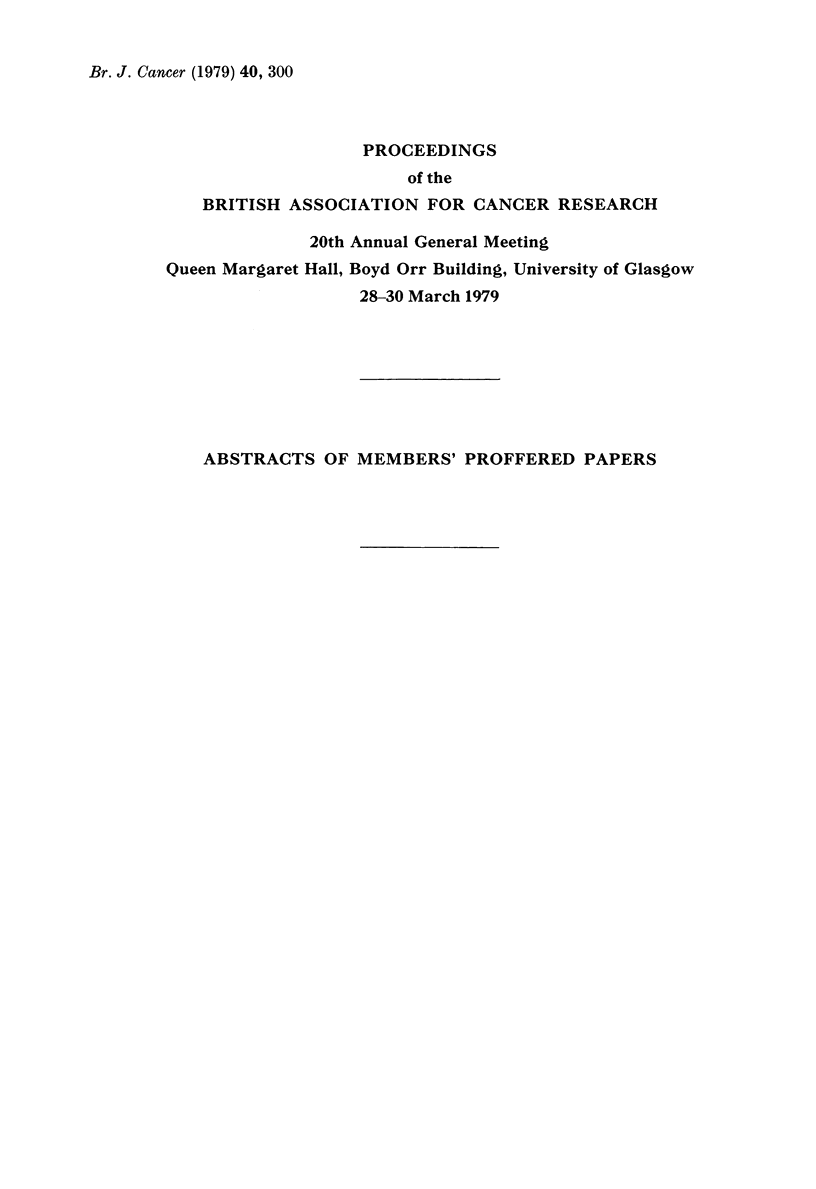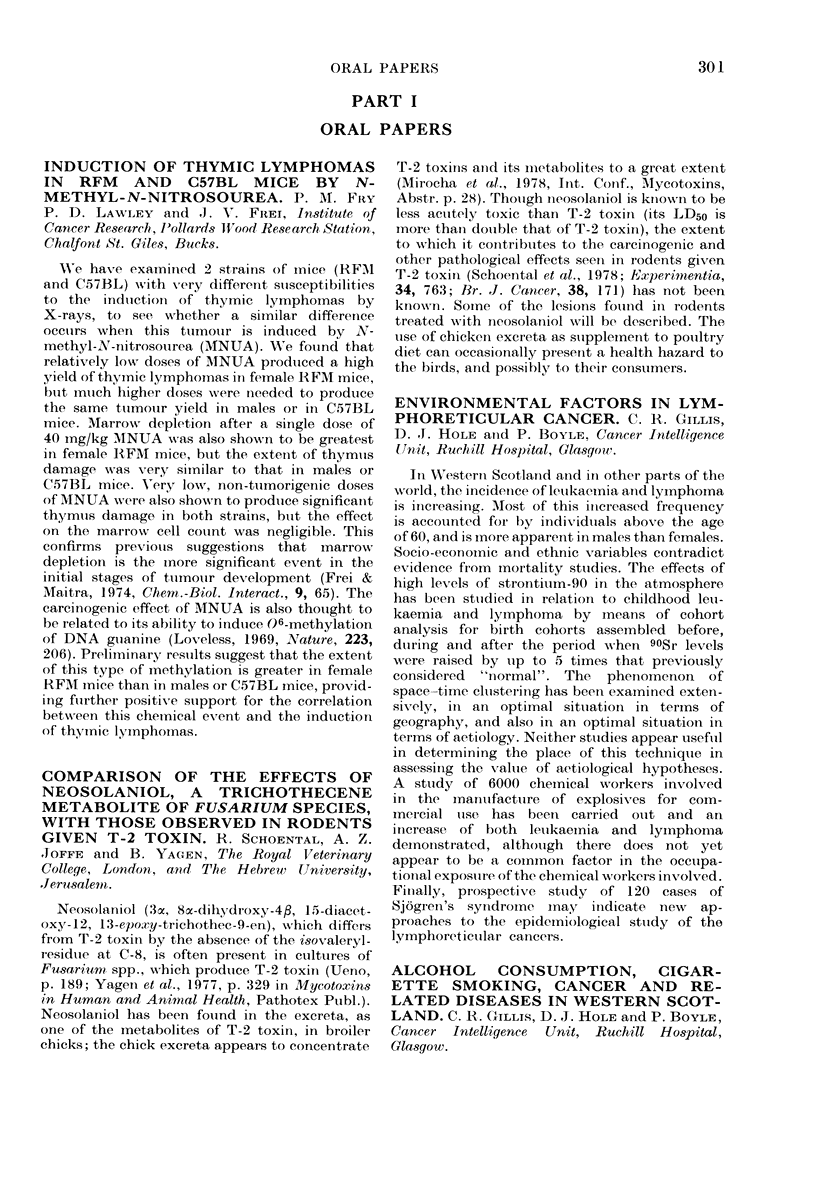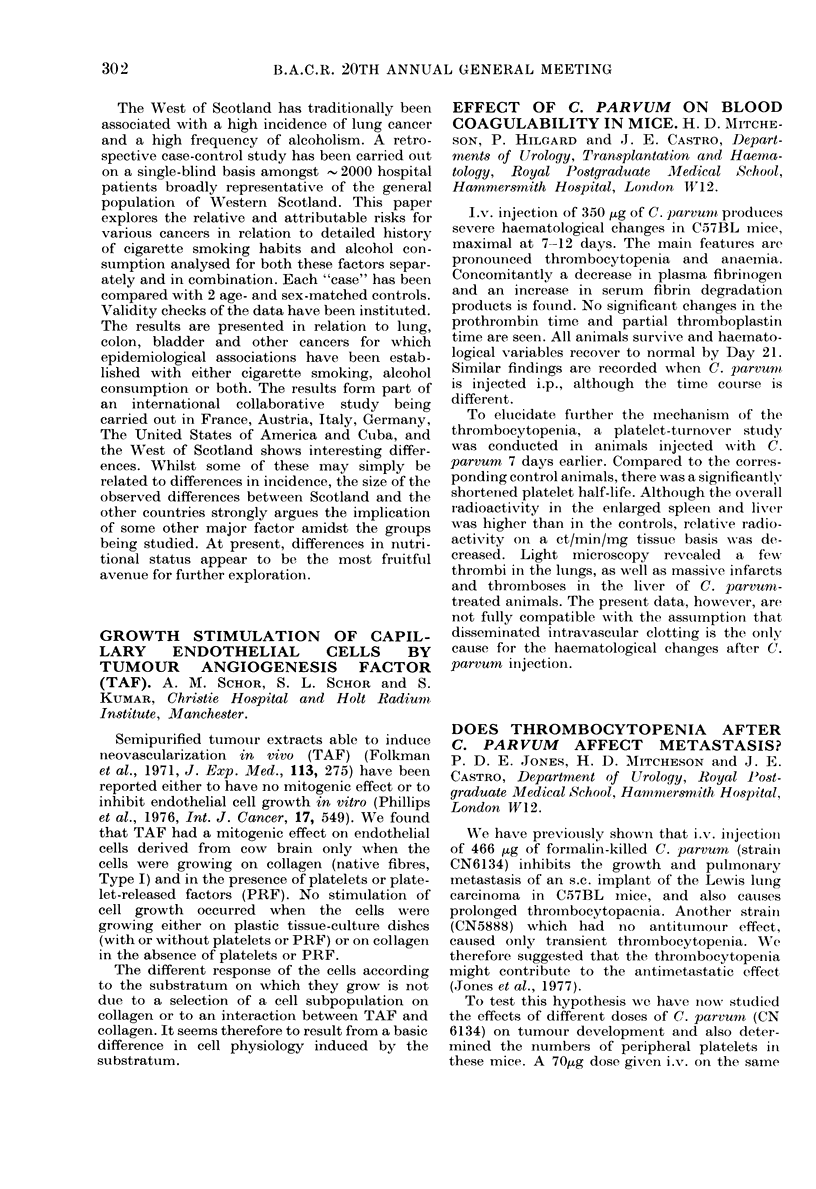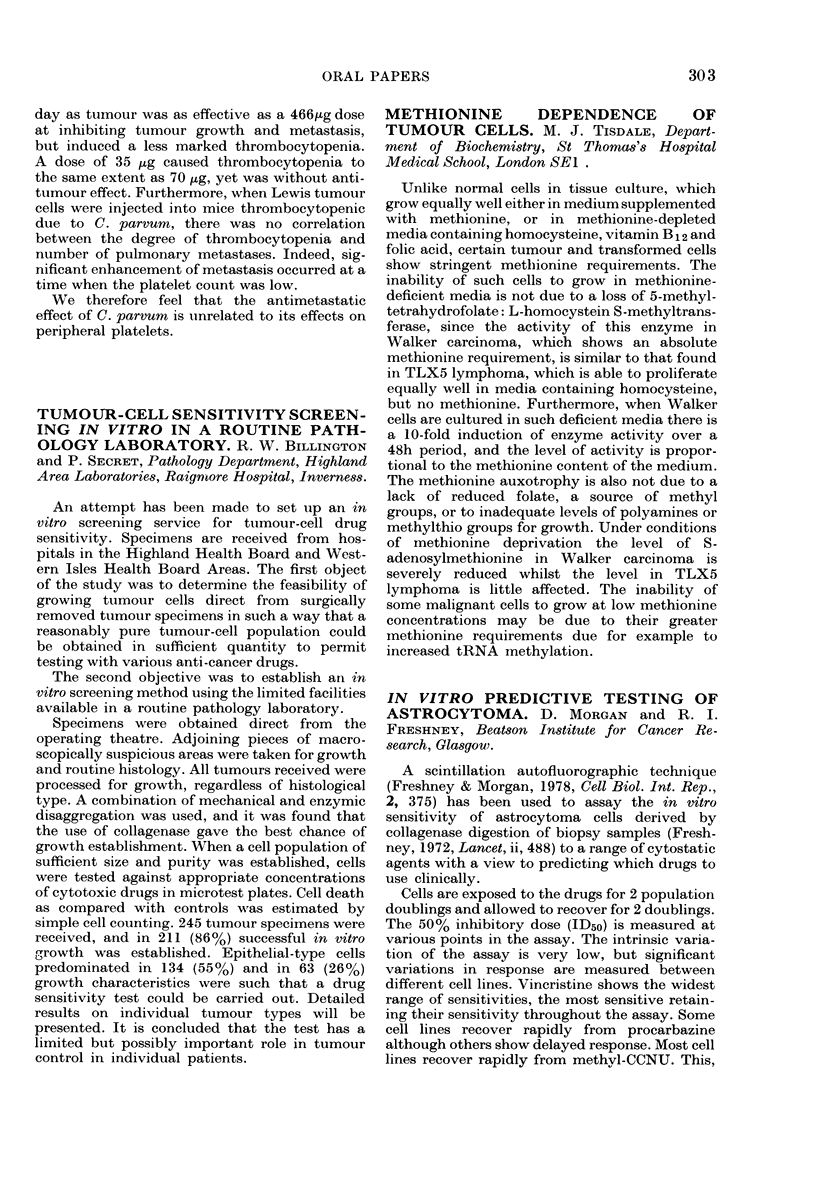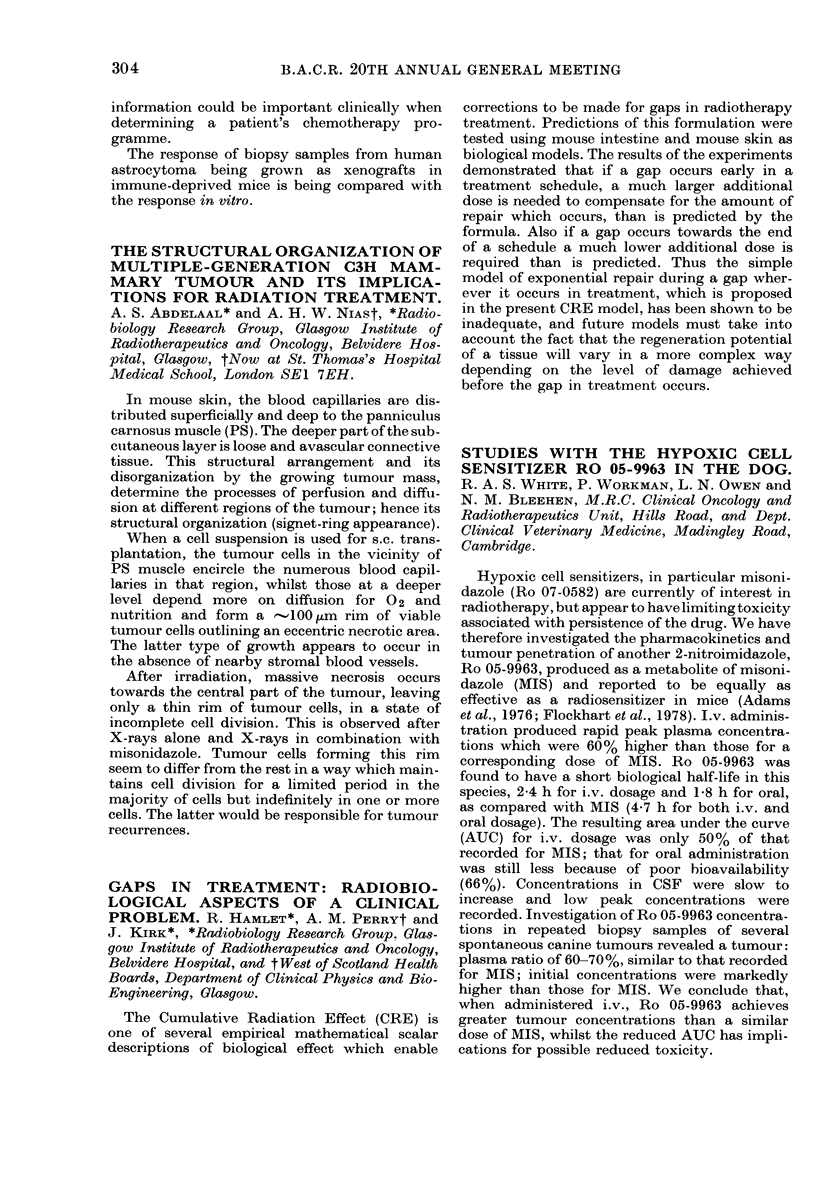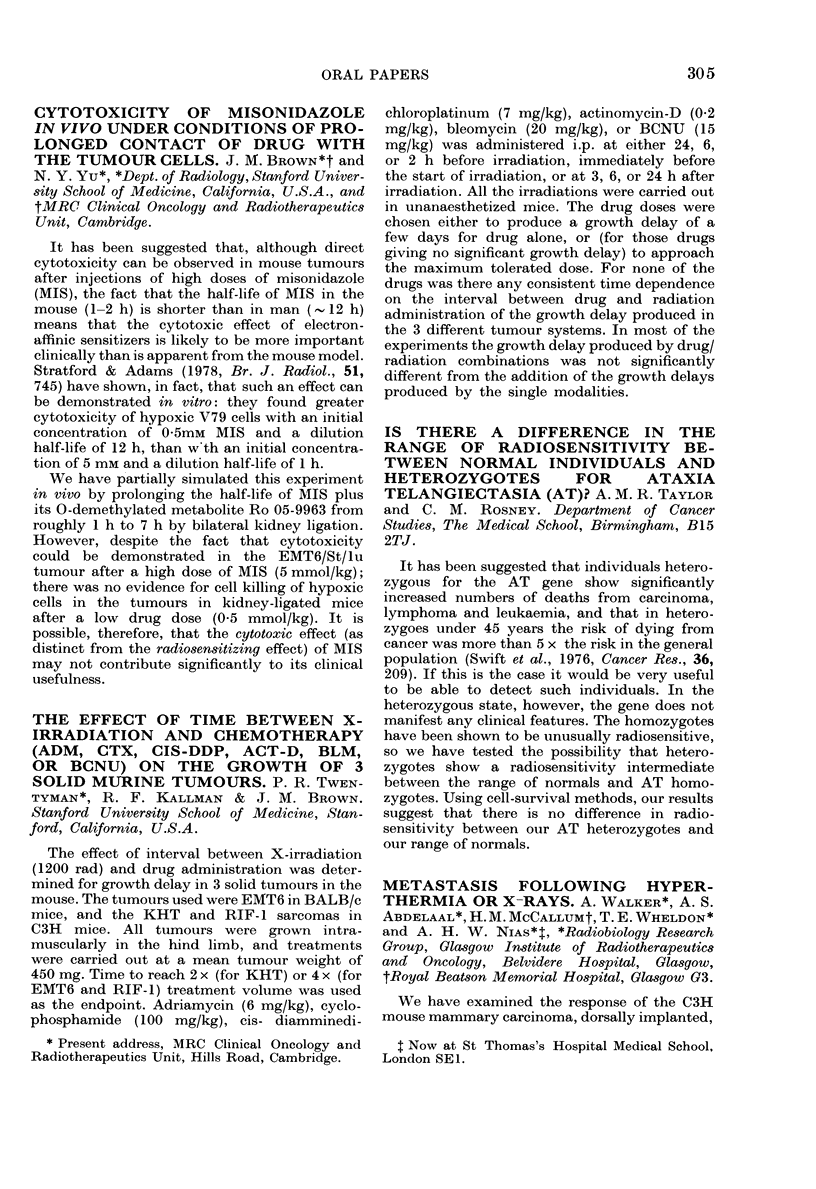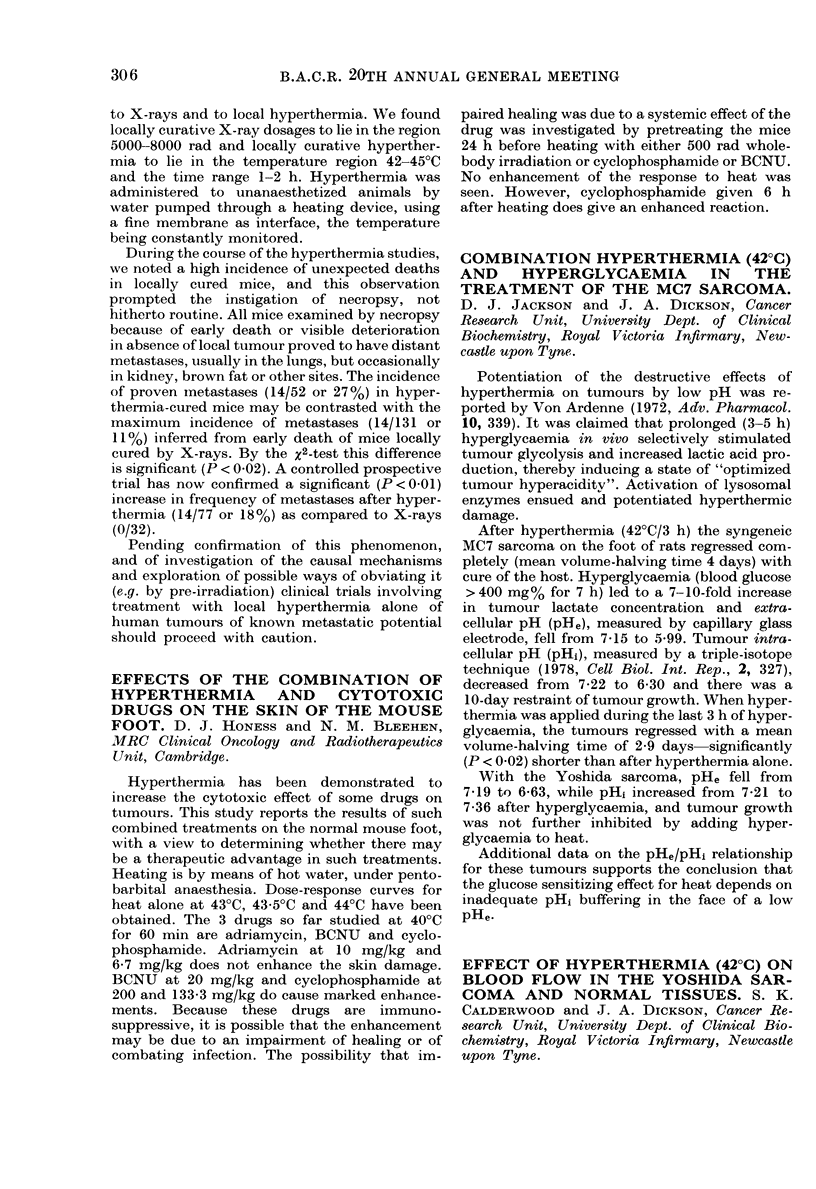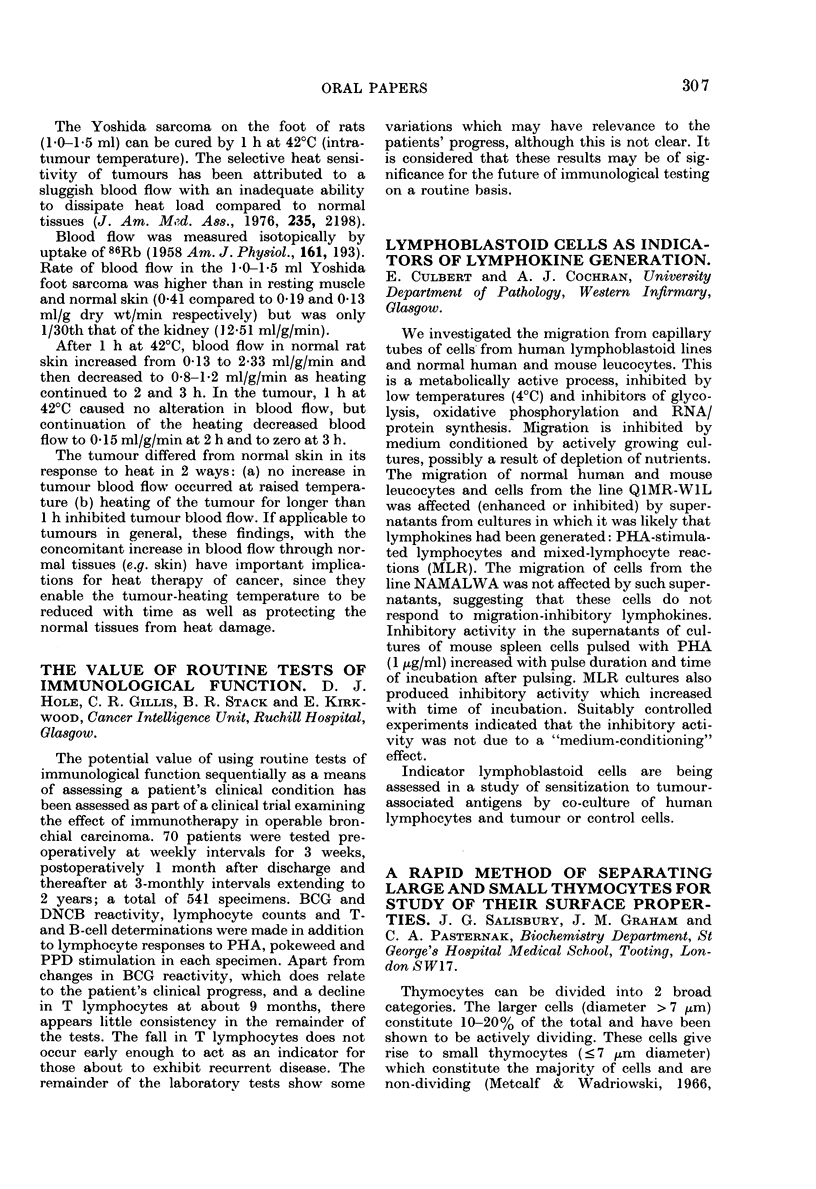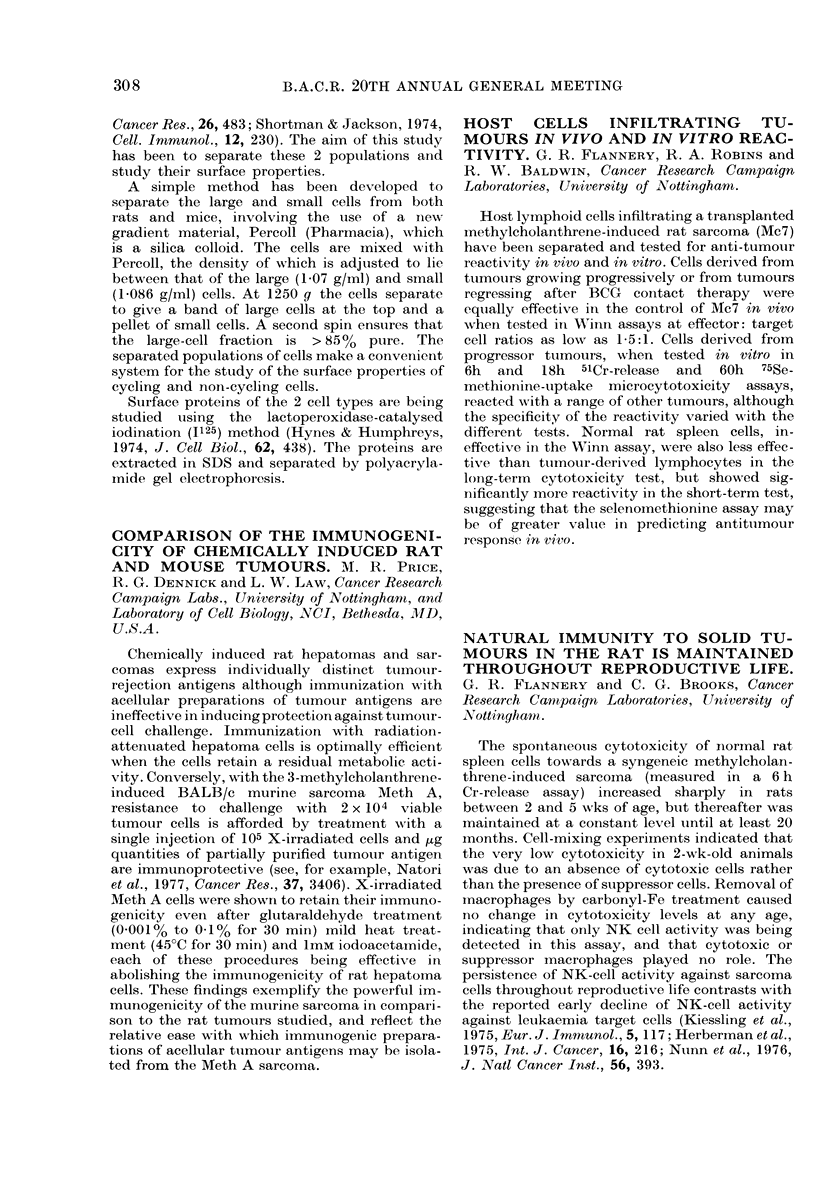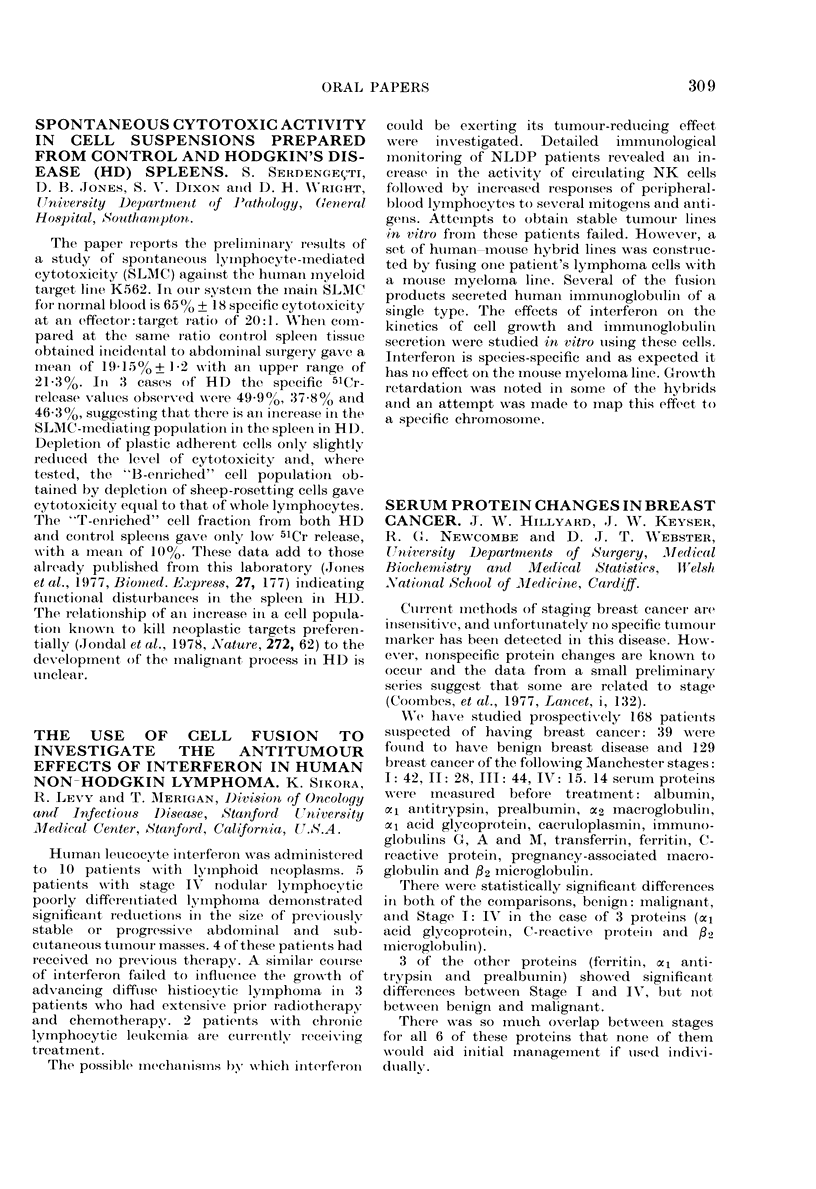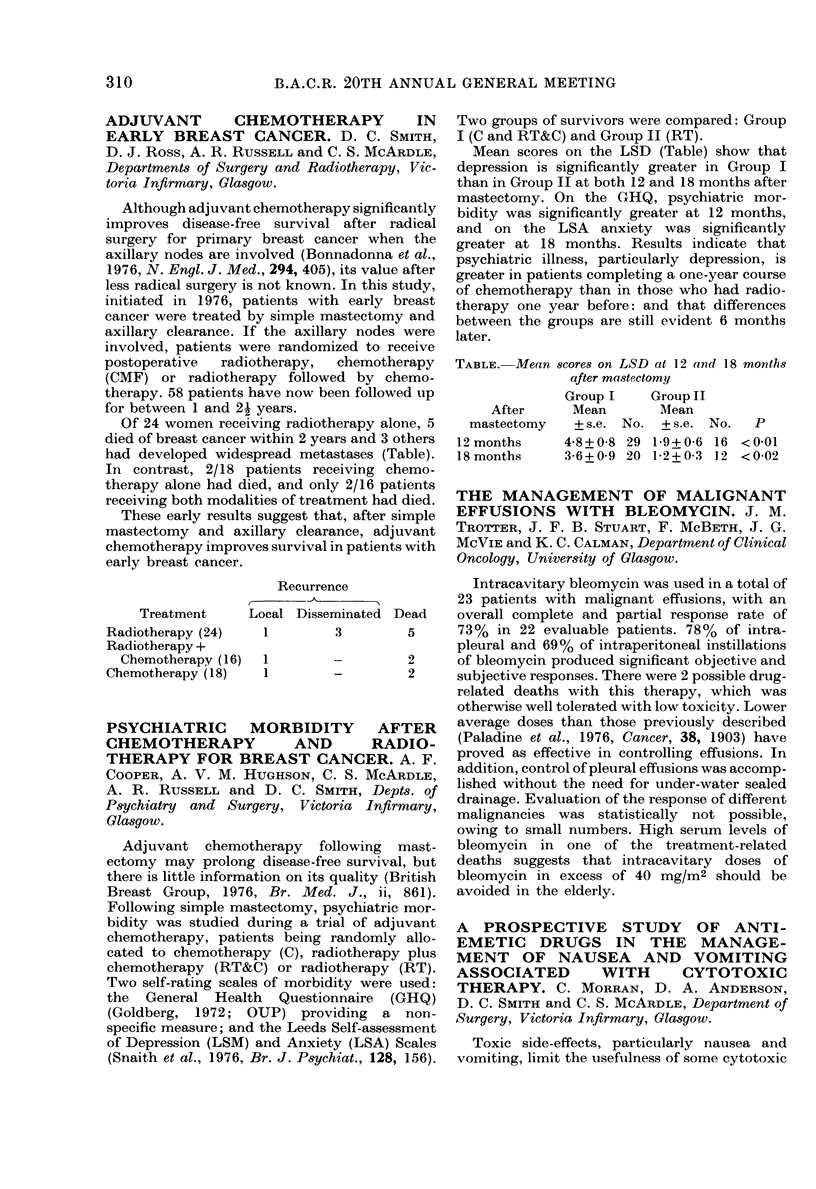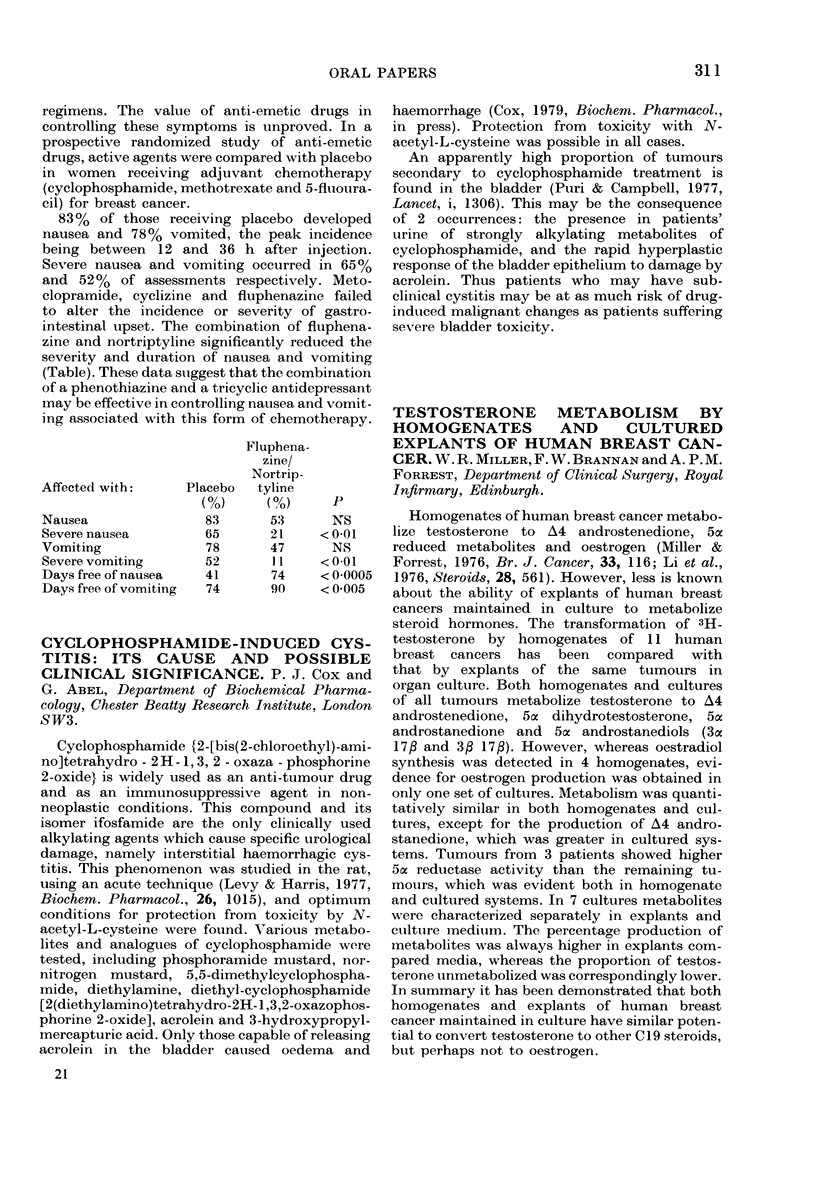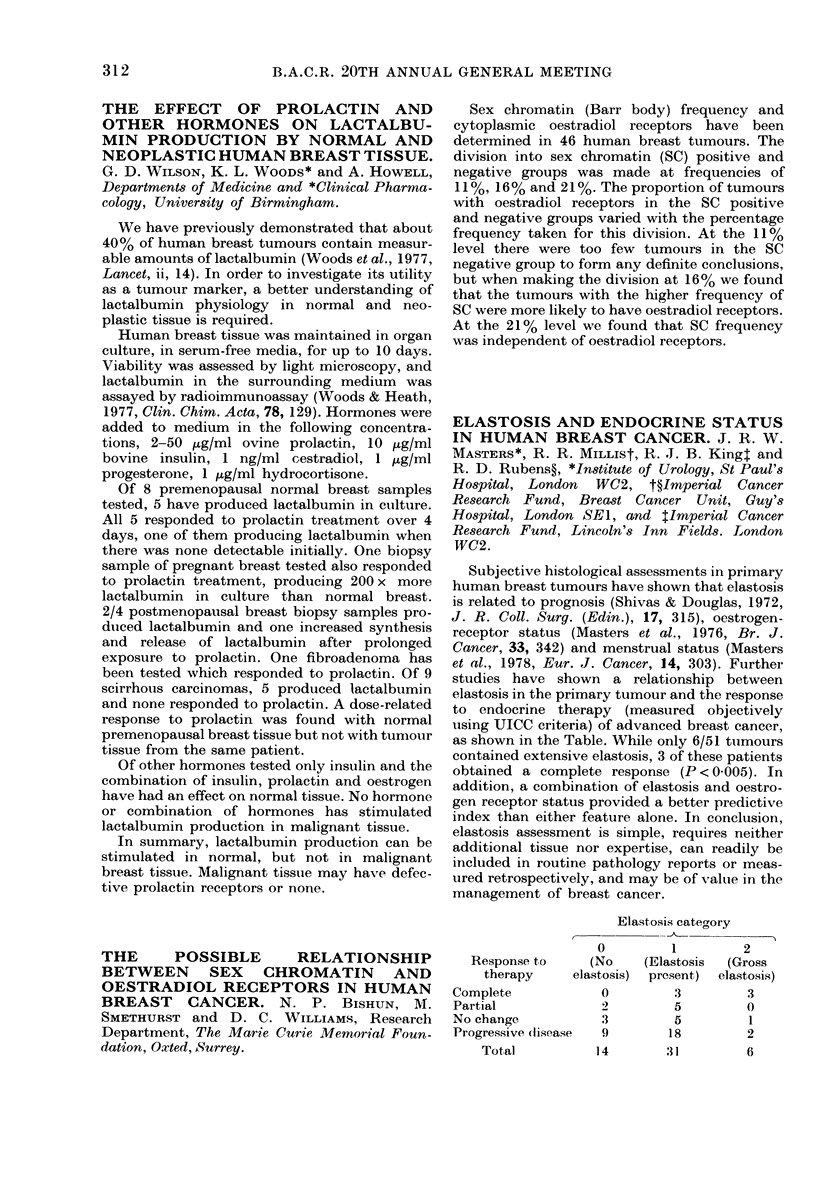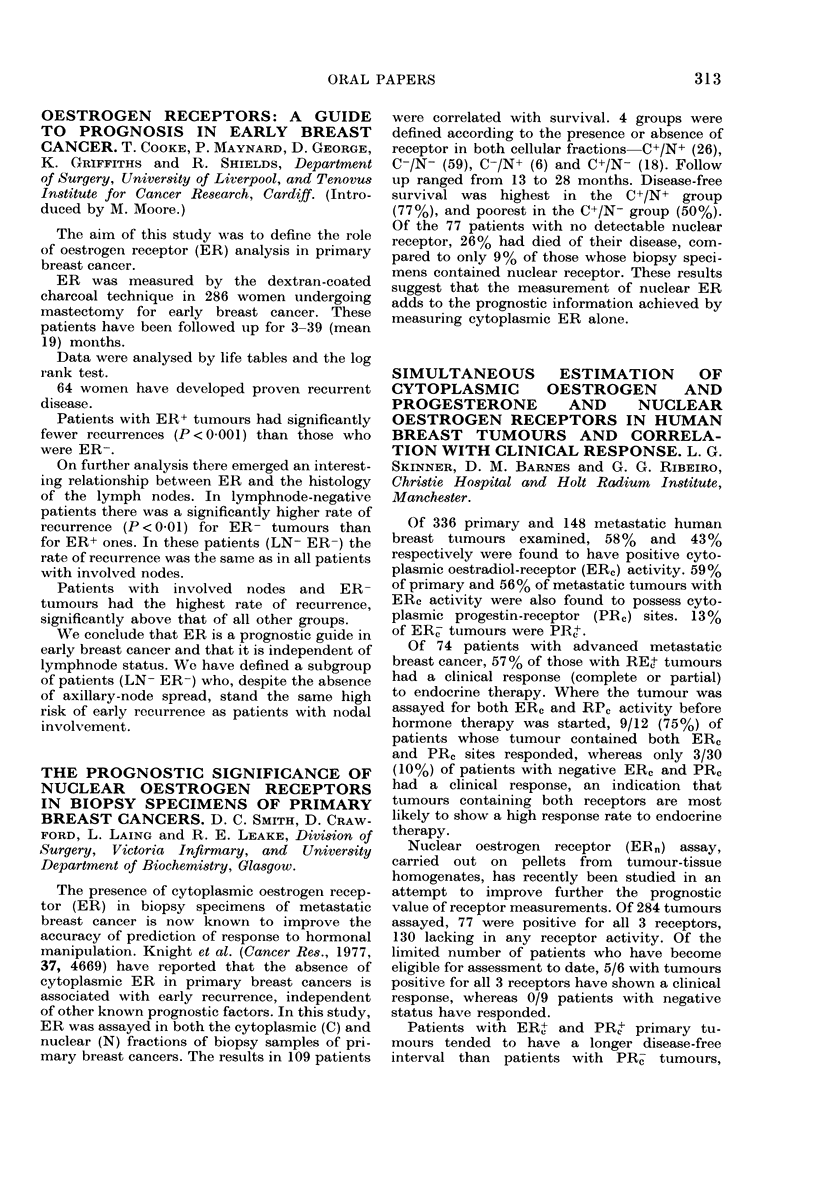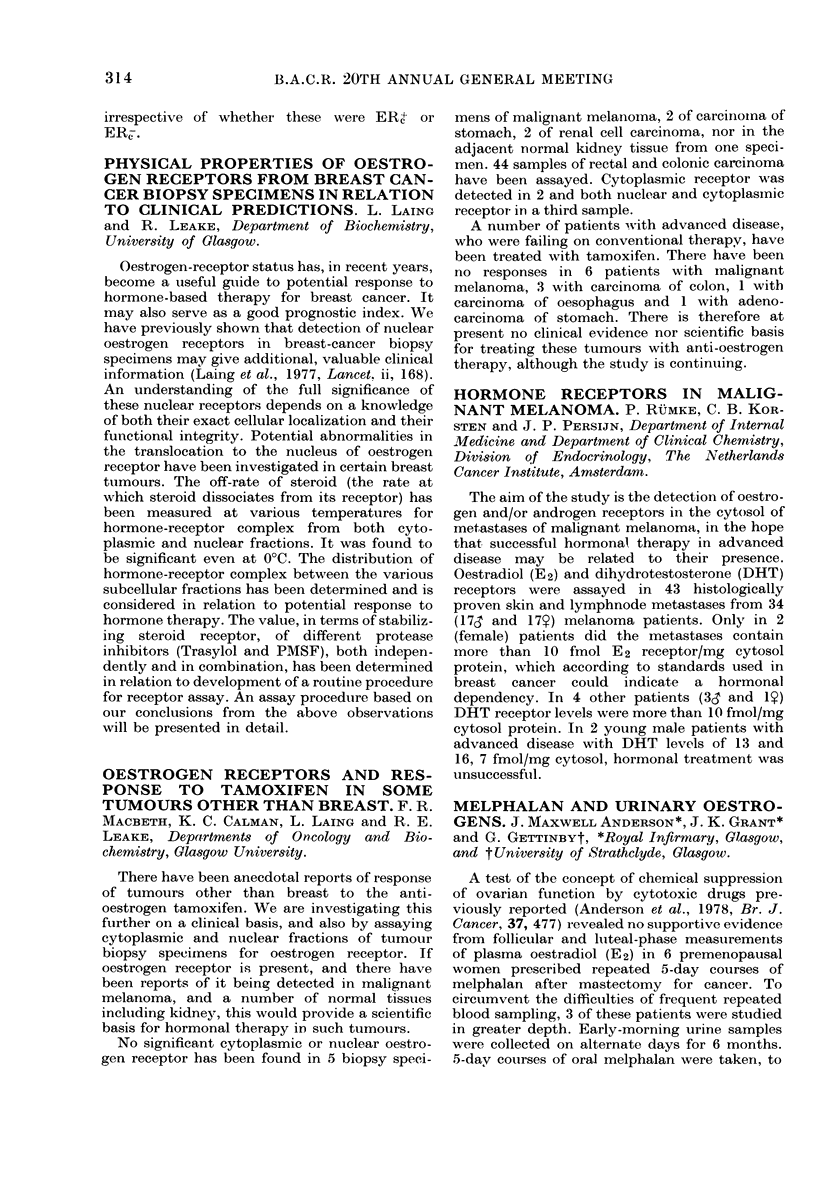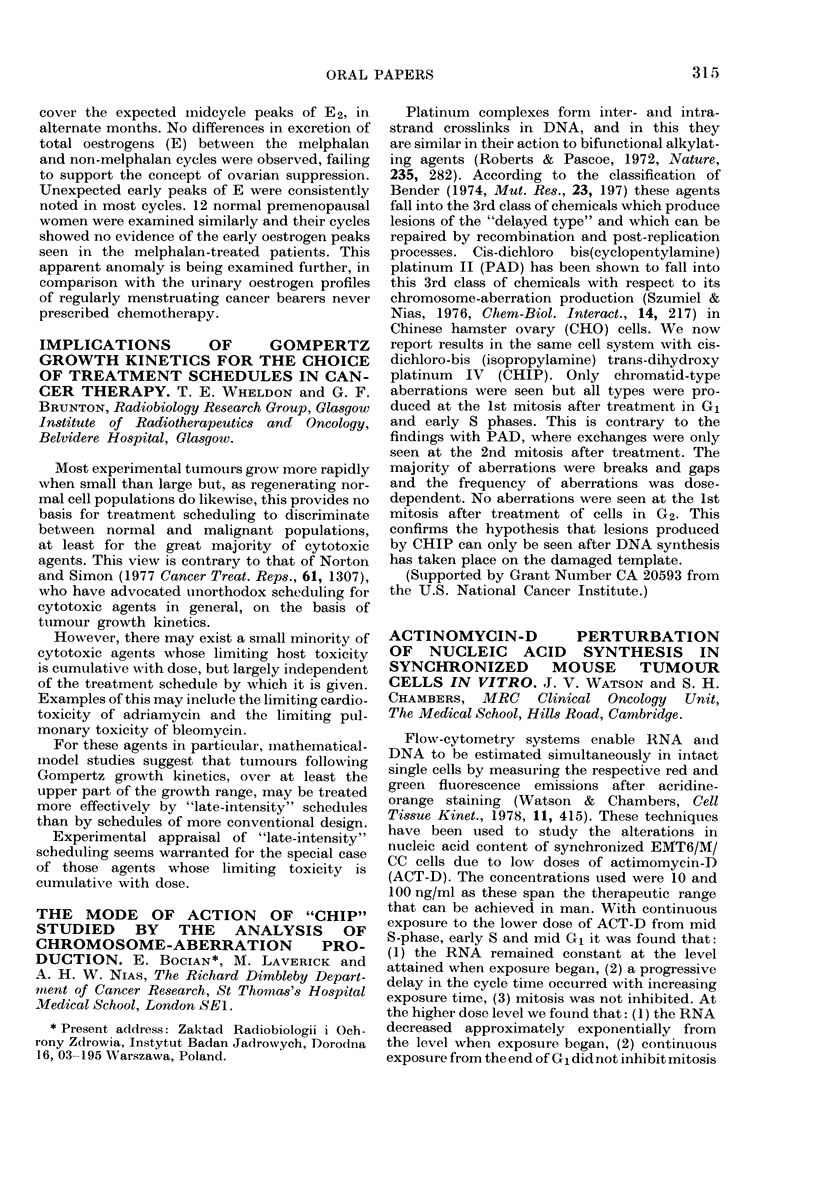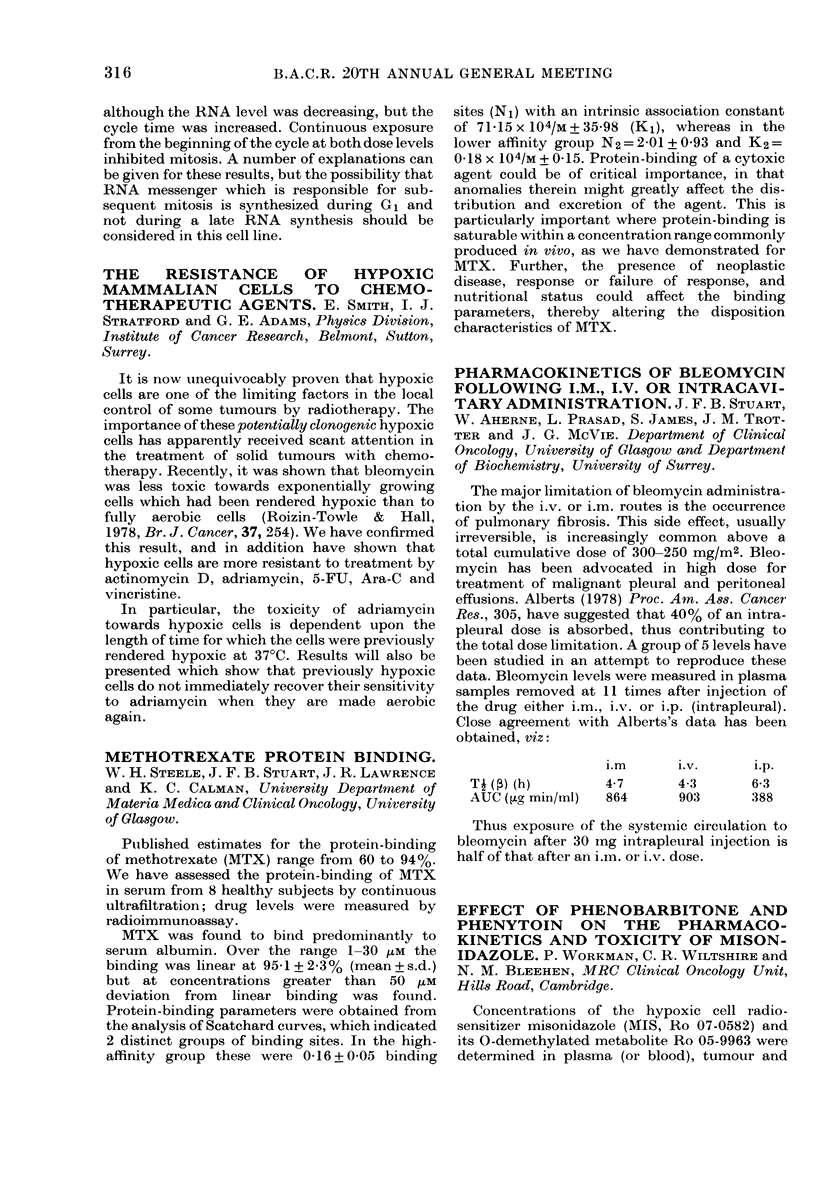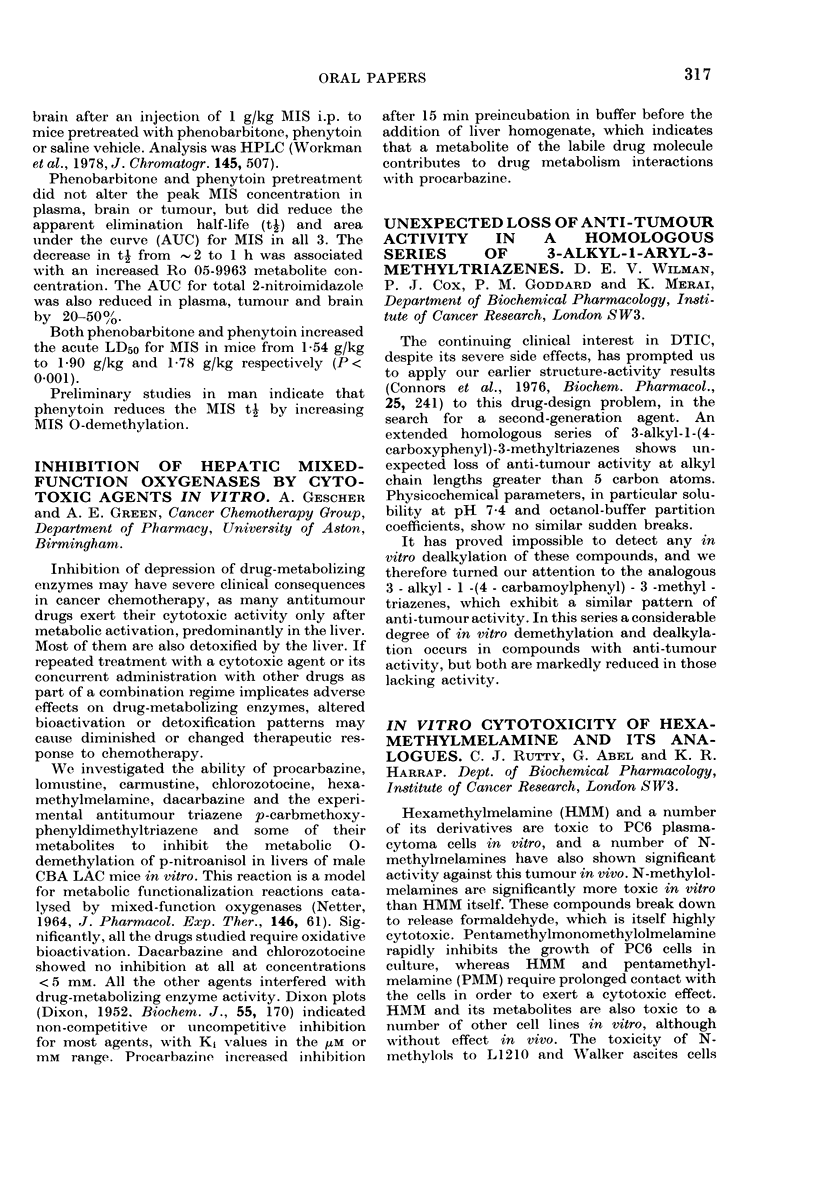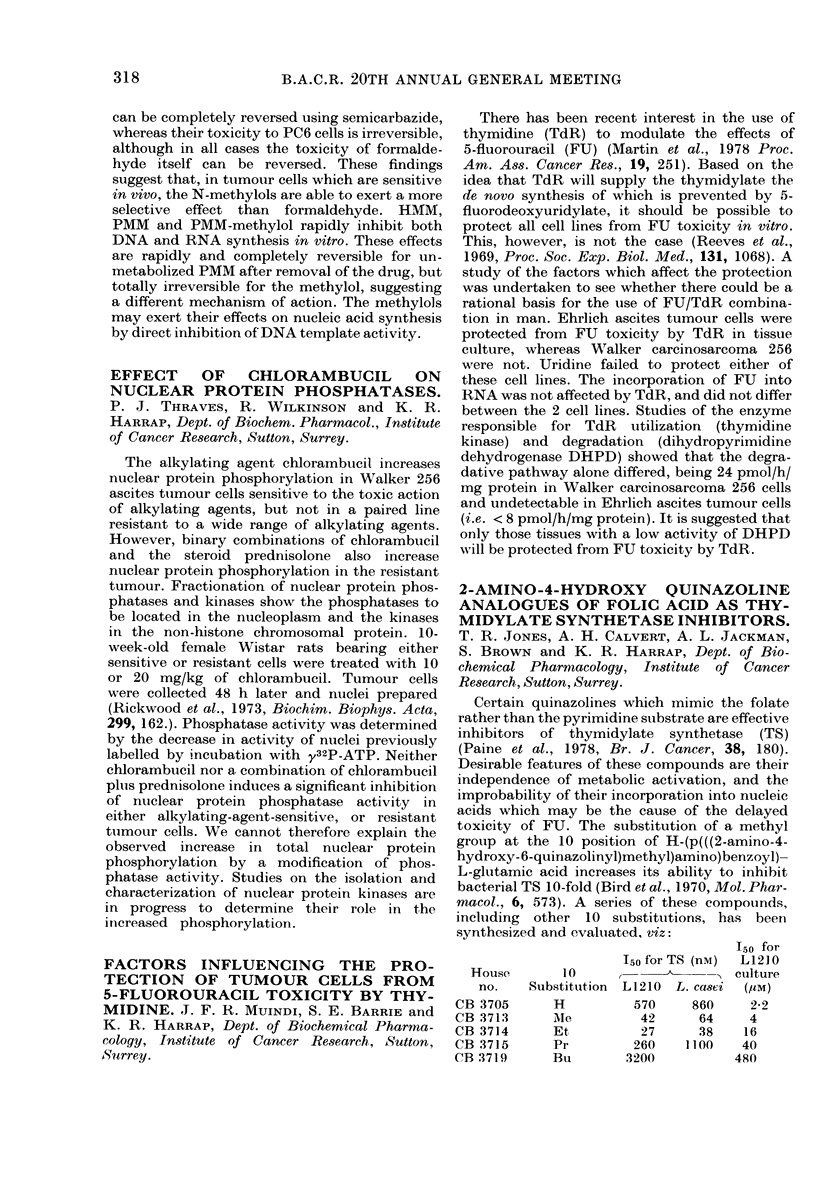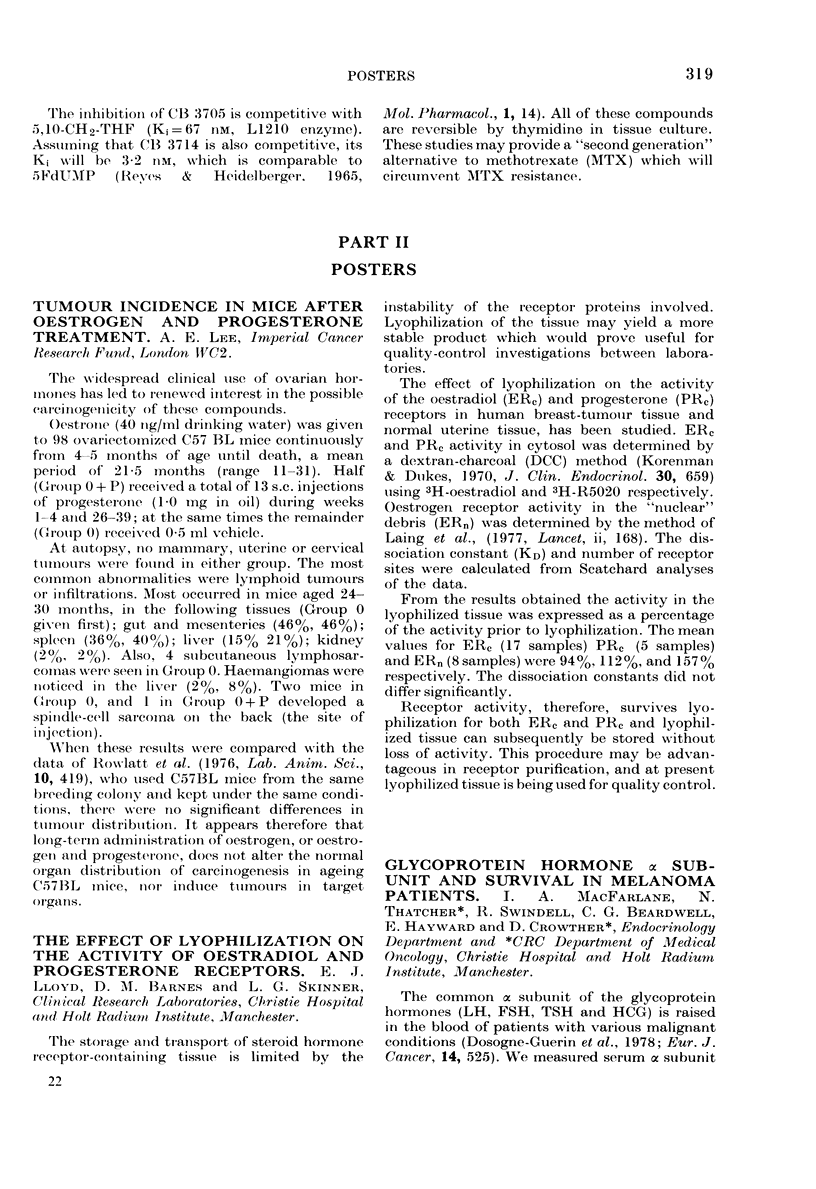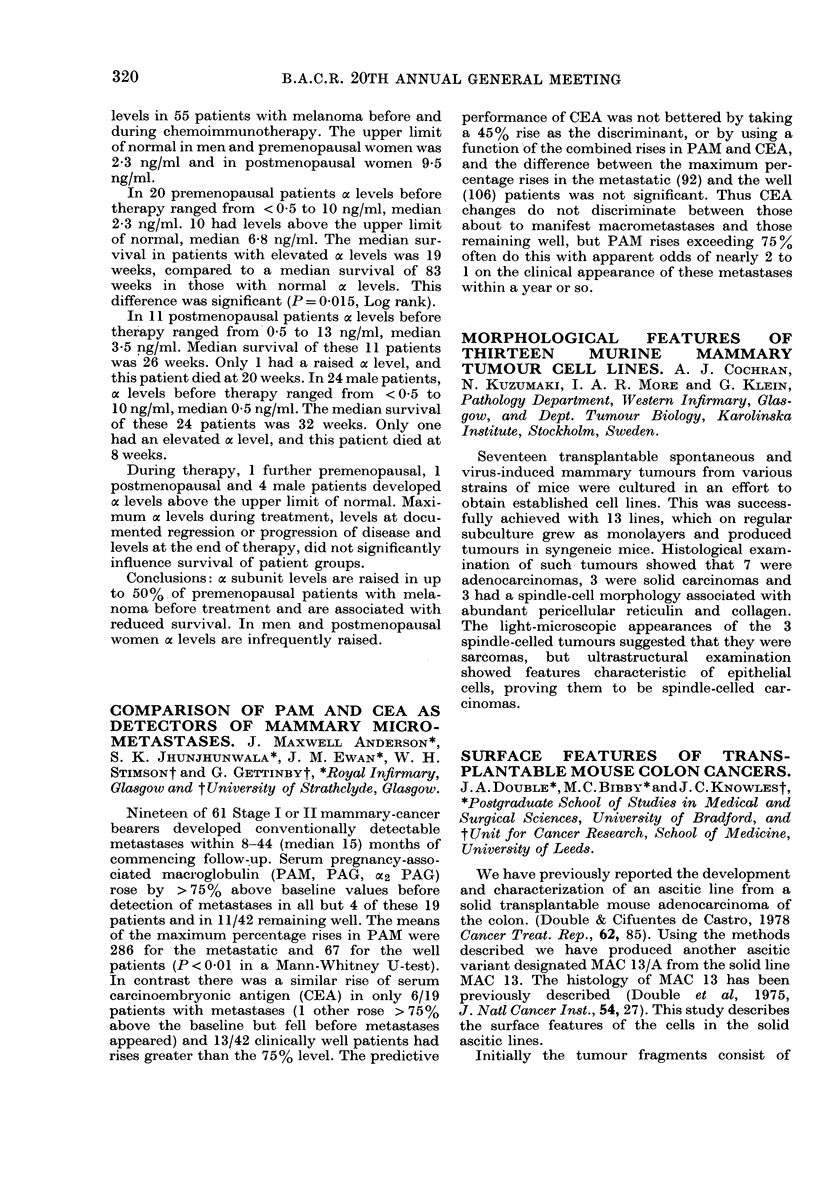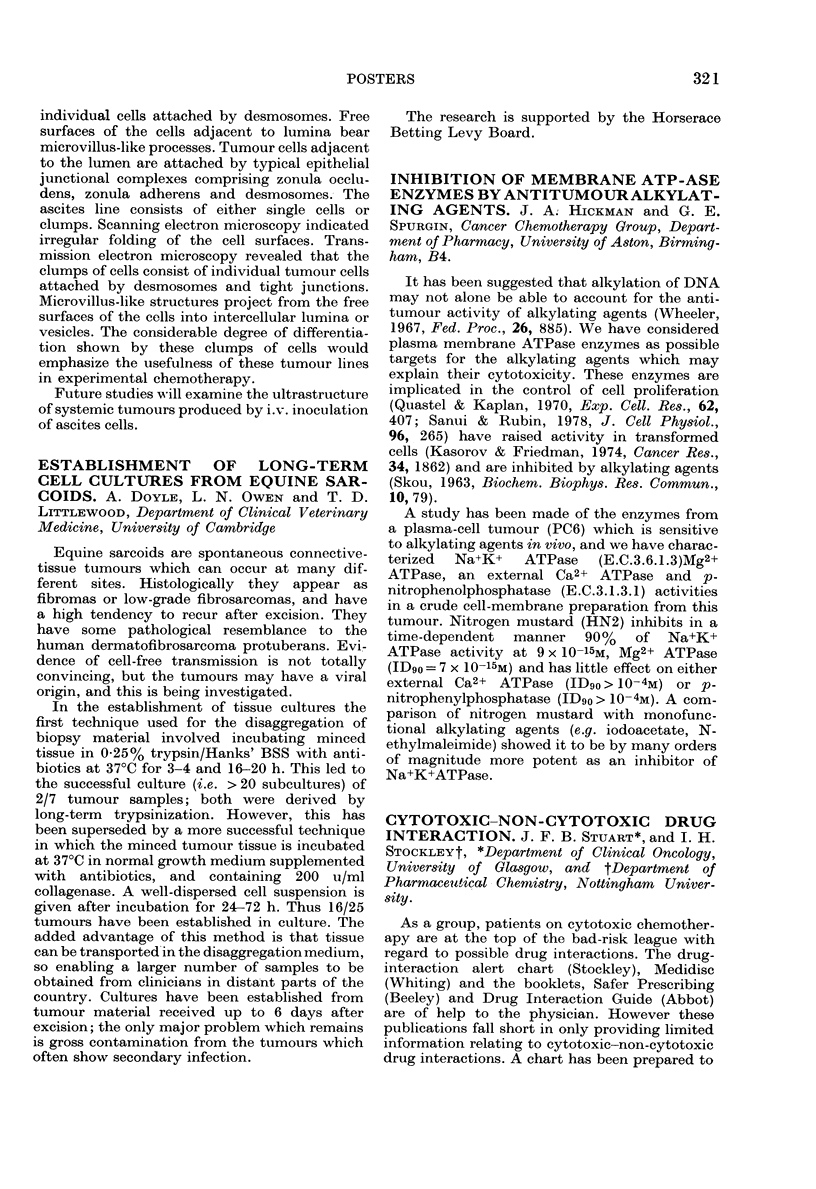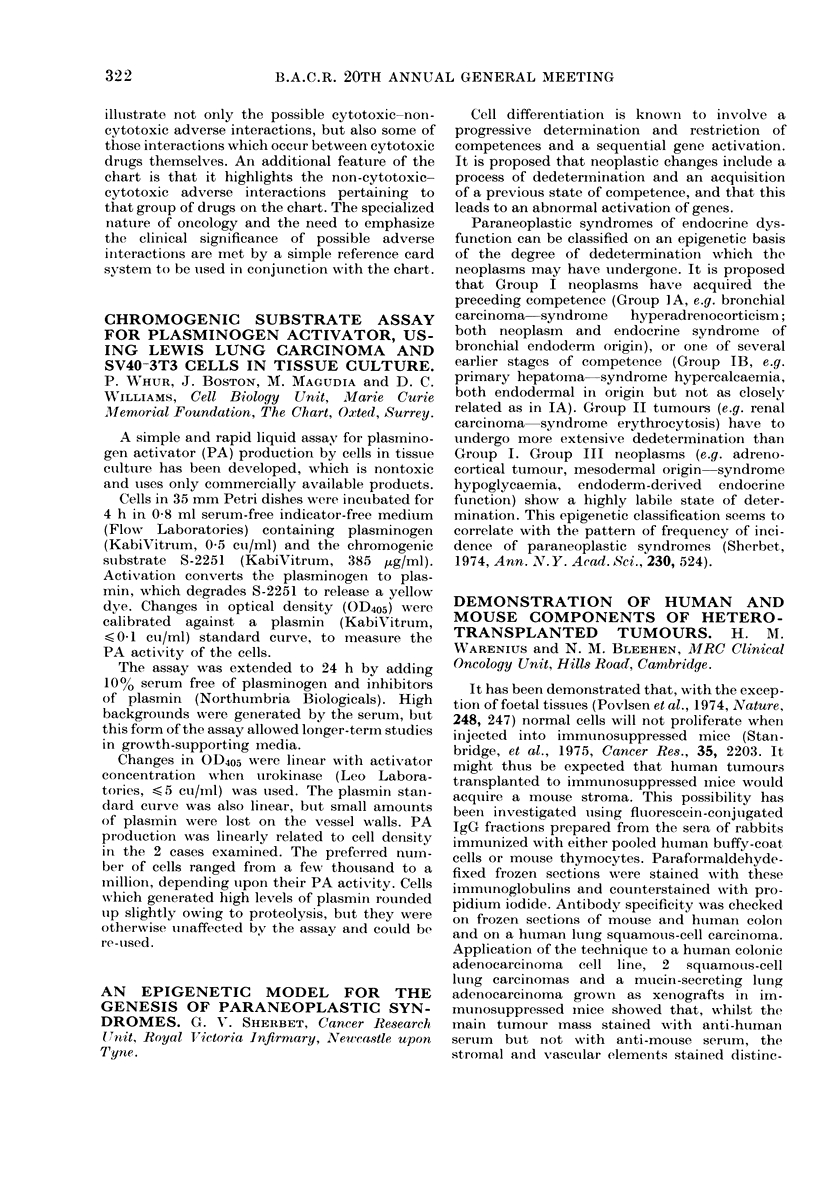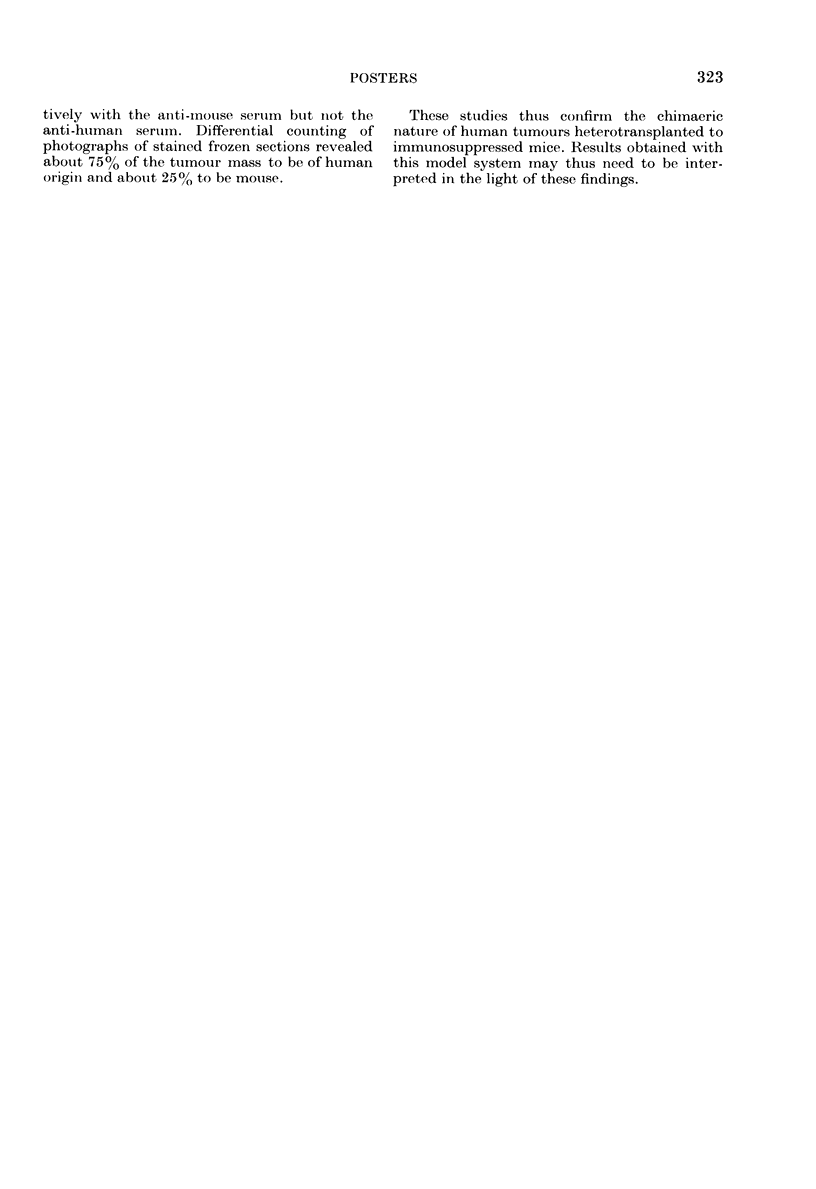# Proceedings of the British Association for Cancer Research. University of Glasgow 28--30 March 1979. Abstracts.

**DOI:** 10.1038/bjc.1979.179

**Published:** 1979-08

**Authors:** 


*Br. J. Cancer* (1979) **40**, 300

### PROCEEDINGS
### of the
### BRITISH ASSOCIATION FOR CANCER RESEARCH

**20th Annual General Meeting**

**Queen Margaret Hall, Boyd Orr Building, University of Glasgow**

**28–30 March 1979**

---

### ABSTRACTS OF MEMBERS' PROFFERED PAPERS

---

### PART I

### ORAL PAPERS

**INDUCTION OF THYMIC LYMPHOMAS IN RFM AND C57BL MICE BY *N*-METHYL-*N*-NITROSOUREA.** P. M. Fry P. D. Lawley and J. V. Frei, *Institute of Cancer Research, Pollards Wood Research Station, Chalfont St. Giles, Bucks.*

We have examined 2 strains of mice (RFM and C57BL) with very different susceptibilities to the induction of thymic lymphomas by X-rays, to see whether a similar difference occurs when this tumour is induced by *N*-methyl-*N*-nitrosourea (MNUA). We found that relatively low doses of MNUA produced a high yield of thymic lymphomas in female RFM mice, but much higher doses were needed to produce the same tumour yield in males or in C57BL mice. Marrow depletion after a single dose of 40 mg/kg MNUA was also shown to be greatest in female RFM mice, but the extent of thymus damage was very similar to that in males or C57BL mice. Very low, non-tumorigenic doses of MNUA were also shown to produce significant thymus damage in both strains, but the effect on the marrow cell count was negligible. This confirms previous suggestions that marrow depletion is the more significant event in the initial stages of tumour development (Frei & Maitra, 1974, *Chem.-Biol. Interact.*, **9**, 65). The carcinogenic effect of MNUA is also thought to be related to its ability to induce $O^6$-methylation of DNA guanine (Loveless, 1969, *Nature*, **223**, 206). Preliminary results suggest that the extent of this type of methylation is greater in female RFM mice than in males or C57BL mice, providing further positive support for the correlation between this chemical event and the induction of thymic lymphomas.

**COMPARISON OF THE EFFECTS OF NEOSOLANIOL, A TRICHOTHECENE METABOLITE OF *FUSARIUM* SPECIES, WITH THOSE OBSERVED IN RODENTS GIVEN T-2 TOXIN.** R. Schoental, A. Z. Joffe and B. Yagen, *The Royal Veterinary College, London, and The Hebrew University, Jerusalem.*

Neosolaniol (3α, 8α-dihydroxy-4β, 15-diacetoxy-12, 13-*epoxy*-trichothec-9-en), which differs from T-2 toxin by the absence of the *iso*valeryl-residue at C-8, is often present in cultures of *Fusarium* spp., which produce T-2 toxin (Ueno, p. 189; Yagen *et al.*, 1977, p. 329 in *Mycotoxins in Human and Animal Health*, Pathotex Publ.). Neosolaniol has been found in the excreta, as one of the metabolites of T-2 toxin, in broiler chicks; the chick excreta appears to concentrate T-2 toxins and its metabolites to a great extent (Mirocha *et al.*, 1978, Int. Conf., Mycotoxins, Abstr. p. 28). Though neosolaniol is known to be less acutely toxic than T-2 toxin (its $LD_{50}$ is more than double that of T-2 toxin), the extent to which it contributes to the carcinogenic and other pathological effects seen in rodents given T-2 toxin (Schoental *et al.*, 1978; *Experimentia*, **34**, 763; *Br. J. Cancer*, **38**, 171) has not been known. Some of the lesions found in rodents treated with neosolaniol will be described. The use of chicken excreta as supplement to poultry diet can occasionally present a health hazard to the birds, and possibly to their consumers.

**ENVIRONMENTAL FACTORS IN LYMPHORETICULAR CANCER.** C. R. Gillis, D. J. Hole and P. Boyle, *Cancer Intelligence Unit, Ruchill Hospital, Glasgow.*

In Western Scotland and in other parts of the world, the incidence of leukaemia and lymphoma is increasing. Most of this increased frequency is accounted for by individuals above the age of 60, and is more apparent in males than females. Socio-economic and ethnic variables contradict evidence from mortality studies. The effects of high levels of strontium-90 in the atmosphere has been studied in relation to childhood leukaemia and lymphoma by means of cohort analysis for birth cohorts assembled before, during and after the period when $^{90}Sr$ levels were raised by up to 5 times that previously considered "normal". The phenomenon of space–time clustering has been examined extensively, in an optimal situation in terms of geography, and also in an optimal situation in terms of aetiology. Neither studies appear useful in determining the place of this technique in assessing the value of aetiological hypotheses. A study of 6000 chemical workers involved in the manufacture of explosives for commercial use has been carried out and an increase of both leukaemia and lymphoma demonstrated, although there does not yet appear to be a common factor in the occupational exposure of the chemical workers involved. Finally, prospective study of 120 cases of Sjögren's syndrome may indicate new approaches to the epidemiological study of the lymphoreticular cancers.

**ALCOHOL CONSUMPTION, CIGARETTE SMOKING, CANCER AND RELATED DISEASES IN WESTERN SCOTLAND.** C. R. Gillis, D. J. Hole and P. Boyle, *Cancer Intelligence Unit, Ruchill Hospital, Glasgow.*

The West of Scotland has traditionally been associated with a high incidence of lung cancer and a high frequency of alcoholism. A retrospective case-control study has been carried out on a single-blind basis amongst ~2000 hospital patients broadly representative of the general population of Western Scotland. This paper explores the relative and attributable risks for various cancers in relation to detailed history of cigarette smoking habits and alcohol consumption analysed for both these factors separately and in combination. Each "case" has been compared with 2 age- and sex-matched controls. Validity checks of the data have been instituted. The results are presented in relation to lung, colon, bladder and other cancers for which epidemiological associations have been established with either cigarette smoking, alcohol consumption or both. The results form part of an international collaborative study being carried out in France, Austria, Italy, Germany, The United States of America and Cuba, and the West of Scotland shows interesting differences. Whilst some of these may simply be related to differences in incidence, the size of the observed differences between Scotland and the other countries strongly argues the implication of some other major factor amidst the groups being studied. At present, differences in nutritional status appear to be the most fruitful avenue for further exploration.

**GROWTH STIMULATION OF CAPILLARY ENDOTHELIAL CELLS BY TUMOUR ANGIOGENESIS FACTOR (TAF).** A. M. Schor, S. L. Schor and S. Kumar, *Christie Hospital and Holt Radium Institute, Manchester.*

Semipurified tumour extracts able to induce neovascularization *in vivo* (TAF) (Folkman *et al.*, 1971, *J. Exp. Med.*, **113,** 275) have been reported either to have no mitogenic effect or to inhibit endothelial cell growth *in vitro* (Phillips *et al.*, 1976, *Int. J. Cancer*, **17,** 549). We found that TAF had a mitogenic effect on endothelial cells derived from cow brain only when the cells were growing on collagen (native fibres, Type I) and in the presence of platelets or platelet-released factors (PRF). No stimulation of cell growth occurred when the cells were growing either on plastic tissue-culture dishes (with or without platelets or PRF) or on collagen in the absence of platelets or PRF.

The different response of the cells according to the substratum on which they grow is not due to a selection of a cell subpopulation on collagen or to an interaction between TAF and collagen. It seems therefore to result from a basic difference in cell physiology induced by the substratum.

**EFFECT OF *C. PARVUM* ON BLOOD COAGULABILITY IN MICE.** H. D. Mitcheson, P. Hilgard and J. E. Castro, *Departments of Urology, Transplantation and Haematology, Royal Postgraduate Medical School, Hammersmith Hospital, London W12.*

I.v. injection of 350 μg of *C. parvum* produces severe haematological changes in C57BL mice, maximal at 7–12 days. The main features are pronounced thrombocytopenia and anaemia. Concomitantly a decrease in plasma fibrinogen and an increase in serum fibrin degradation products is found. No significant changes in the prothrombin time and partial thromboplastin time are seen. All animals survive and haematological variables recover to normal by Day 21. Similar findings are recorded when *C. parvum* is injected i.p., although the time course is different.

To elucidate further the mechanism of the thrombocytopenia, a platelet-turnover study was conducted in animals injected with *C. parvum* 7 days earlier. Compared to the corresponding control animals, there was a significantly shortened platelet half-life. Although the overall radioactivity in the enlarged spleen and liver was higher than in the controls, relative radioactivity on a ct/min/mg tissue basis was decreased. Light microscopy revealed a few thrombi in the lungs, as well as massive infarcts and thromboses in the liver of *C. parvum*-treated animals. The present data, however, are not fully compatible with the assumption that disseminated intravascular clotting is the only cause for the haematological changes after *C. parvum* injection.

**DOES THROMBOCYTOPENIA AFTER *C. PARVUM* AFFECT METASTASIS?** P. D. E. Jones, H. D. Mitcheson and J. E. Castro, *Department of Urology, Royal Postgraduate Medical School, Hammersmith Hospital, London W12.*

We have previously shown that i.v. injection of 466 μg of formalin-killed *C. parvum* (strain CN6134) inhibits the growth and pulmonary metastasis of an s.c. implant of the Lewis lung carcinoma in C57BL mice, and also causes prolonged thrombocytopaenia. Another strain (CN5888) which had no antitumour effect, caused only transient thrombocytopenia. We therefore suggested that the thrombocytopenia might contribute to the antimetastatic effect (Jones *et al.*, 1977).

To test this hypothesis we have now studied the effects of different doses of *C. parvum* (CN 6134) on tumour development and also determined the numbers of peripheral platelets in these mice. A 70μg dose given i.v. on the same day as tumour was as effective as a 466μg dose at inhibiting tumour growth and metastasis, but induced a less marked thrombocytopenia. A dose of 35 μg caused thrombocytopenia to the same extent as 70 μg, yet was without antitumour effect. Furthermore, when Lewis tumour cells were injected into mice thrombocytopenic due to *C. parvum*, there was no correlation between the degree of thrombocytopenia and number of pulmonary metastases. Indeed, significant enhancement of metastasis occurred at a time when the platelet count was low.

We therefore feel that the antimetastatic effect of *C. parvum* is unrelated to its effects on peripheral platelets.

**TUMOUR-CELL SENSITIVITY SCREENING *IN VITRO* IN A ROUTINE PATHOLOGY LABORATORY.** R. W. BILLINGTON and P. SECRET, *Pathology Department, Highland Area Laboratories, Raigmore Hospital, Inverness.*

An attempt has been made to set up an *in vitro* screening service for tumour-cell drug sensitivity. Specimens are received from hospitals in the Highland Health Board and Western Isles Health Board Areas. The first object of the study was to determine the feasibility of growing tumour cells direct from surgically removed tumour specimens in such a way that a reasonably pure tumour-cell population could be obtained in sufficient quantity to permit testing with various anti-cancer drugs.

The second objective was to establish an *in vitro* screening method using the limited facilities available in a routine pathology laboratory.

Specimens were obtained direct from the operating theatre. Adjoining pieces of macroscopically suspicious areas were taken for growth and routine histology. All tumours received were processed for growth, regardless of histological type. A combination of mechanical and enzymic disaggregation was used, and it was found that the use of collagenase gave the best chance of growth establishment. When a cell population of sufficient size and purity was established, cells were tested against appropriate concentrations of cytotoxic drugs in microtest plates. Cell death as compared with controls was estimated by simple cell counting. 245 tumour specimens were received, and in 211 (86%) successful *in vitro* growth was established. Epithelial-type cells predominated in 134 (55%) and in 63 (26%) growth characteristics were such that a drug sensitivity test could be carried out. Detailed results on individual tumour types will be presented. It is concluded that the test has a limited but possibly important role in tumour control in individual patients.

**METHIONINE DEPENDENCE OF TUMOUR CELLS.** M. J. TISDALE, *Department of Biochemistry, St Thomas's Hospital Medical School, London SE1* .

Unlike normal cells in tissue culture, which grow equally well either in medium supplemented with methionine, or in methionine-depleted media containing homocysteine, vitamin $B_{12}$ and folic acid, certain tumour and transformed cells show stringent methionine requirements. The inability of such cells to grow in methionine-deficient media is not due to a loss of 5-methyltetrahydrofolate: L-homocystein S-methyltransferase, since the activity of this enzyme in Walker carcinoma, which shows an absolute methionine requirement, is similar to that found in TLX5 lymphoma, which is able to proliferate equally well in media containing homocysteine, but no methionine. Furthermore, when Walker cells are cultured in such deficient media there is a 10-fold induction of enzyme activity over a 48h period, and the level of activity is proportional to the methionine content of the medium. The methionine auxotrophy is also not due to a lack of reduced folate, a source of methyl groups, or to inadequate levels of polyamines or methylthio groups for growth. Under conditions of methionine deprivation the level of S-adenosylmethionine in Walker carcinoma is severely reduced whilst the level in TLX5 lymphoma is little affected. The inability of some malignant cells to grow at low methionine concentrations may be due to their greater methionine requirements due for example to increased tRNA methylation.

***IN VITRO* PREDICTIVE TESTING OF ASTROCYTOMA.** D. MORGAN and R. I. FRESHNEY, *Beatson Institute for Cancer Research, Glasgow.*

A scintillation autofluorographic technique (Freshney & Morgan, 1978, *Cell Biol. Int. Rep.*, **2**, 375) has been used to assay the *in vitro* sensitivity of astrocytoma cells derived by collagenase digestion of biopsy samples (Freshney, 1972, *Lancet*, ii, 488) to a range of cytostatic agents with a view to predicting which drugs to use clinically.

Cells are exposed to the drugs for 2 population doublings and allowed to recover for 2 doublings. The 50% inhibitory dose ($ID_{50}$) is measured at various points in the assay. The intrinsic variation of the assay is very low, but significant variations in response are measured between different cell lines. Vincristine shows the widest range of sensitivities, the most sensitive retaining their sensitivity throughout the assay. Some cell lines recover rapidly from procarbazine although others show delayed response. Most cell lines recover rapidly from methyl-CCNU. This, information could be important clinically when determining a patient's chemotherapy programme.

The response of biopsy samples from human astrocytoma being grown as xenografts in immune-deprived mice is being compared with the response *in vitro*.

**THE STRUCTURAL ORGANIZATION OF MULTIPLE-GENERATION C3H MAMMARY TUMOUR AND ITS IMPLICATIONS FOR RADIATION TREATMENT.** A. S. ABDELAAL* and A. H. W. NIAS†, **Radiobiology Research Group, Glasgow Institute of Radiotherapeutics and Oncology, Belvidere Hospital, Glasgow,* †*Now at St. Thomas's Hospital Medical School, London SE1 7EH.*

In mouse skin, the blood capillaries are distributed superficially and deep to the panniculus carnosus muscle (PS). The deeper part of the subcutaneous layer is loose and avascular connective tissue. This structural arrangement and its disorganization by the growing tumour mass, determine the processes of perfusion and diffusion at different regions of the tumour; hence its structural organization (signet-ring appearance).

When a cell suspension is used for s.c. transplantation, the tumour cells in the vicinity of PS muscle encircle the numerous blood capillaries in that region, whilst those at a deeper level depend more on diffusion for $O_2$ and nutrition and form a $\sim 100\,\mu m$ rim of viable tumour cells outlining an eccentric necrotic area. The latter type of growth appears to occur in the absence of nearby stromal blood vessels.

After irradiation, massive necrosis occurs towards the central part of the tumour, leaving only a thin rim of tumour cells, in a state of incomplete cell division. This is observed after X-rays alone and X-rays in combination with misonidazole. Tumour cells forming this rim seem to differ from the rest in a way which maintains cell division for a limited period in the majority of cells but indefinitely in one or more cells. The latter would be responsible for tumour recurrences.

**GAPS IN TREATMENT: RADIOBIOLOGICAL ASPECTS OF A CLINICAL PROBLEM.** R. HAMLET*, A. M. PERRY† and J. KIRK*, **Radiobiology Research Group, Glasgow Institute of Radiotherapeutics and Oncology, Belvidere Hospital, and* †*West of Scotland Health Boards, Department of Clinical Physics and Bio-Engineering, Glasgow.*

The Cumulative Radiation Effect (CRE) is one of several empirical mathematical scalar descriptions of biological effect which enable corrections to be made for gaps in radiotherapy treatment. Predictions of this formulation were tested using mouse intestine and mouse skin as biological models. The results of the experiments demonstrated that if a gap occurs early in a treatment schedule, a much larger additional dose is needed to compensate for the amount of repair which occurs, than is predicted by the formula. Also if a gap occurs towards the end of a schedule a much lower additional dose is required than is predicted. Thus the simple model of exponential repair during a gap wherever it occurs in treatment, which is proposed in the present CRE model, has been shown to be inadequate, and future models must take into account the fact that the regeneration potential of a tissue will vary in a more complex way depending on the level of damage achieved before the gap in treatment occurs.

**STUDIES WITH THE HYPOXIC CELL SENSITIZER RO 05-9963 IN THE DOG.** R. A. S. WHITE, P. WORKMAN, L. N. OWEN and N. M. BLEEHEN, *M.R.C. Clinical Oncology and Radiotherapeutics Unit, Hills Road, and Dept. Clinical Veterinary Medicine, Madingley Road, Cambridge.*

Hypoxic cell sensitizers, in particular misonidazole (Ro 07-0582) are currently of interest in radiotherapy, but appear to have limiting toxicity associated with persistence of the drug. We have therefore investigated the pharmacokinetics and tumour penetration of another 2-nitroimidazole, Ro 05-9963, produced as a metabolite of misonidazole (MIS) and reported to be equally as effective as a radiosensitizer in mice (Adams *et al.*, 1976; Flockhart *et al.*, 1978). I.v. administration produced rapid peak plasma concentrations which were 60% higher than those for a corresponding dose of MIS. Ro 05-9963 was found to have a short biological half-life in this species, 2·4 h for i.v. dosage and 1·8 h for oral, as compared with MIS (4·7 h for both i.v. and oral dosage). The resulting area under the curve (AUC) for i.v. dosage was only 50% of that recorded for MIS; that for oral administration was still less because of poor bioavailability (66%). Concentrations in CSF were slow to increase and low peak concentrations were recorded. Investigation of Ro 05-9963 concentrations in repeated biopsy samples of several spontaneous canine tumours revealed a tumour: plasma ratio of 60–70%, similar to that recorded for MIS; initial concentrations were markedly higher than those for MIS. We conclude that, when administered i.v., Ro 05-9963 achieves greater tumour concentrations than a similar dose of MIS, whilst the reduced AUC has implications for possible reduced toxicity.

**CYTOTOXICITY OF MISONIDAZOLE *IN VIVO* UNDER CONDITIONS OF PROLONGED CONTACT OF DRUG WITH THE TUMOUR CELLS.** J. M. Brown*† and N. Y. Yu*, **Dept. of Radiology, Stanford University School of Medicine, California, U.S.A., and †MRC Clinical Oncology and Radiotherapeutics Unit, Cambridge.*

It has been suggested that, although direct cytotoxicity can be observed in mouse tumours after injections of high doses of misonidazole (MIS), the fact that the half-life of MIS in the mouse (1–2 h) is shorter than in man ($\sim$12 h) means that the cytotoxic effect of electron-affinic sensitizers is likely to be more important clinically than is apparent from the mouse model. Stratford & Adams (1978, *Br. J. Radiol.*, **51**, 745) have shown, in fact, that such an effect can be demonstrated *in vitro*: they found greater cytotoxicity of hypoxic V79 cells with an initial concentration of 0·5mM MIS and a dilution half-life of 12 h, than w'th an initial concentration of 5 mM and a dilution half-life of 1 h.

We have partially simulated this experiment *in vivo* by prolonging the half-life of MIS plus its O-demethylated metabolite Ro 05-9963 from roughly 1 h to 7 h by bilateral kidney ligation. However, despite the fact that cytotoxicity could be demonstrated in the EMT6/St/1u tumour after a high dose of MIS (5 mmol/kg); there was no evidence for cell killing of hypoxic cells in the tumours in kidney-ligated mice after a low drug dose (0·5 mmol/kg). It is possible, therefore, that the *cytotoxic* effect (as distinct from the *radiosensitizing* effect) of MIS may not contribute significantly to its clinical usefulness.

**THE EFFECT OF TIME BETWEEN X-IRRADIATION AND CHEMOTHERAPY (ADM, CTX, CIS-DDP, ACT-D, BLM, OR BCNU) ON THE GROWTH OF 3 SOLID MURINE TUMOURS.** P. R. Twentyman*, R. F. Kallman & J. M. Brown. *Stanford University School of Medicine, Stanford, California, U.S.A.*

The effect of interval between X-irradiation (1200 rad) and drug administration was determined for growth delay in 3 solid tumours in the mouse. The tumours used were EMT6 in BALB/c mice, and the KHT and RIF-1 sarcomas in C3H mice. All tumours were grown intramuscularly in the hind limb, and treatments were carried out at a mean tumour weight of 450 mg. Time to reach 2× (for KHT) or 4× (for EMT6 and RIF-1) treatment volume was used as the endpoint. Adriamycin (6 mg/kg), cyclophosphamide (100 mg/kg), cis- diamminedichloroplatinum (7 mg/kg), actinomycin-D (0·2 mg/kg), bleomycin (20 mg/kg), or BCNU (15 mg/kg) was administered i.p. at either 24, 6, or 2 h before irradiation, immediately before the start of irradiation, or at 3, 6, or 24 h after irradiation. All the irradiations were carried out in unanaesthetized mice. The drug doses were chosen either to produce a growth delay of a few days for drug alone, or (for those drugs giving no significant growth delay) to approach the maximum tolerated dose. For none of the drugs was there any consistent time dependence on the interval between drug and radiation administration of the growth delay produced in the 3 different tumour systems. In most of the experiments the growth delay produced by drug/radiation combinations was not significantly different from the addition of the growth delays produced by the single modalities.

* Present address, MRC Clinical Oncology and Radiotherapeutics Unit, Hills Road, Cambridge.

**IS THERE A DIFFERENCE IN THE RANGE OF RADIOSENSITIVITY BETWEEN NORMAL INDIVIDUALS AND HETEROZYGOTES FOR ATAXIA TELANGIECTASIA (AT)?** A. M. R. Taylor and C. M. Rosney. *Department of Cancer Studies, The Medical School, Birmingham, B15 2TJ.*

It has been suggested that individuals heterozygous for the AT gene show significantly increased numbers of deaths from carcinoma, lymphoma and leukaemia, and that in heterozygoes under 45 years the risk of dying from cancer was more than 5× the risk in the general population (Swift *et al.*, 1976, *Cancer Res.*, **36**, 209). If this is the case it would be very useful to be able to detect such individuals. In the heterozygous state, however, the gene does not manifest any clinical features. The homozygotes have been shown to be unusually radiosensitive, so we have tested the possibility that heterozygotes show a radiosensitivity intermediate between the range of normals and AT homozygotes. Using cell-survival methods, our results suggest that there is no difference in radiosensitivity between our AT heterozygotes and our range of normals.

**METASTASIS FOLLOWING HYPERTHERMIA OR X-RAYS.** A. Walker*, A. S. Abdelaal*, H. M. McCallum†, T. E. Wheldon* and A. H. W. Nias*‡, **Radiobiology Research Group, Glasgow Institute of Radiotherapeutics and Oncology, Belvidere Hospital, Glasgow, †Royal Beatson Memorial Hospital, Glasgow G3.*

We have examined the response of the C3H mouse mammary carcinoma, dorsally implanted,

‡ Now at St Thomas's Hospital Medical School, London SE1.

to X-rays and to local hyperthermia. We found locally curative X-ray dosages to lie in the region 5000–8000 rad and locally curative hyperthermia to lie in the temperature region 42–45°C and the time range 1–2 h. Hyperthermia was administered to unanaesthetized animals by water pumped through a heating device, using a fine membrane as interface, the temperature being constantly monitored.

During the course of the hyperthermia studies, we noted a high incidence of unexpected deaths in locally cured mice, and this observation prompted the instigation of necropsy, not hitherto routine. All mice examined by necropsy because of early death or visible deterioration in absence of local tumour proved to have distant metastases, usually in the lungs, but occasionally in kidney, brown fat or other sites. The incidence of proven metastases (14/52 or 27%) in hyperthermia-cured mice may be contrasted with the maximum incidence of metastases (14/131 or 11%) inferred from early death of mice locally cured by X-rays. By the $\chi^2$-test this difference is significant ($P < 0{\cdot}02$). A controlled prospective trial has now confirmed a significant ($P < 0{\cdot}01$) increase in frequency of metastases after hyperthermia (14/77 or 18%) as compared to X-rays (0/32).

Pending confirmation of this phenomenon, and of investigation of the causal mechanisms and exploration of possible ways of obviating it (*e.g.* by pre-irradiation) clinical trials involving treatment with local hyperthermia alone of human tumours of known metastatic potential should proceed with caution.

### EFFECTS OF THE COMBINATION OF HYPERTHERMIA AND CYTOTOXIC DRUGS ON THE SKIN OF THE MOUSE FOOT.

D. J. HONESS and N. M. BLEEHEN, *MRC Clinical Oncology and Radiotherapeutics Unit, Cambridge.*

Hyperthermia has been demonstrated to increase the cytotoxic effect of some drugs on tumours. This study reports the results of such combined treatments on the normal mouse foot, with a view to determining whether there may be a therapeutic advantage in such treatments. Heating is by means of hot water, under pentobarbital anaesthesia. Dose-response curves for heat alone at 43°C, 43·5°C and 44°C have been obtained. The 3 drugs so far studied at 40°C for 60 min are adriamycin, BCNU and cyclophosphamide. Adriamycin at 10 mg/kg and 6·7 mg/kg does not enhance the skin damage. BCNU at 20 mg/kg and cyclophosphamide at 200 and 133·3 mg/kg do cause marked enhancements. Because these drugs are immunosuppressive, it is possible that the enhancement may be due to an impairment of healing or of combating infection. The possibility that impaired healing was due to a systemic effect of the drug was investigated by pretreating the mice 24 h before heating with either 500 rad whole-body irradiation or cyclophosphamide or BCNU. No enhancement of the response to heat was seen. However, cyclophosphamide given 6 h after heating does give an enhanced reaction.

### COMBINATION HYPERTHERMIA (42°C) AND HYPERGLYCAEMIA IN THE TREATMENT OF THE MC7 SARCOMA.

D. J. JACKSON and J. A. DICKSON, *Cancer Research Unit, University Dept. of Clinical Biochemistry, Royal Victoria Infirmary, Newcastle upon Tyne.*

Potentiation of the destructive effects of hyperthermia on tumours by low pH was reported by Von Ardenne (1972, *Adv. Pharmacol.* **10,** 339). It was claimed that prolonged (3–5 h) hyperglycaemia *in vivo* selectively stimulated tumour glycolysis and increased lactic acid production, thereby inducing a state of "optimized tumour hyperacidity". Activation of lysosomal enzymes ensued and potentiated hyperthermic damage.

After hyperthermia (42°C/3 h) the syngeneic MC7 sarcoma on the foot of rats regressed completely (mean volume-halving time 4 days) with cure of the host. Hyperglycaemia (blood glucose >400 mg% for 7 h) led to a 7–10-fold increase in tumour lactate concentration and *extra*cellular pH ($pH_e$), measured by capillary glass electrode, fell from 7·15 to 5·99. Tumour *intra*cellular pH ($pH_i$), measured by a triple-isotope technique (1978, *Cell Biol. Int. Rep.*, **2,** 327), decreased from 7·22 to 6·30 and there was a 10-day restraint of tumour growth. When hyperthermia was applied during the last 3 h of hyperglycaemia, the tumours regressed with a mean volume-halving time of 2·9 days—significantly ($P < 0{\cdot}02$) shorter than after hyperthermia alone.

With the Yoshida sarcoma, $pH_e$ fell from 7·19 to 6·63, while $pH_i$ increased from 7·21 to 7·36 after hyperglycaemia, and tumour growth was not further inhibited by adding hyperglycaemia to heat.

Additional data on the $pH_e/pH_i$ relationship for these tumours supports the conclusion that the glucose sensitizing effect for heat depends on inadequate $pH_i$ buffering in the face of a low $pH_e$.

### EFFECT OF HYPERTHERMIA (42°C) ON BLOOD FLOW IN THE YOSHIDA SARCOMA AND NORMAL TISSUES.

S. K. CALDERWOOD and J. A. DICKSON, *Cancer Research Unit, University Dept. of Clinical Biochemistry, Royal Victoria Infirmary, Newcastle upon Tyne.*

The Yoshida sarcoma on the foot of rats (1·0–1·5 ml) can be cured by 1 h at 42°C (intratumour temperature). The selective heat sensitivity of tumours has been attributed to a sluggish blood flow with an inadequate ability to dissipate heat load compared to normal tissues (*J. Am. Med. Ass.*, 1976, **235**, 2198).

Blood flow was measured isotopically by uptake of $^{86}$Rb (1958 *Am. J. Physiol.*, **161**, 193). Rate of blood flow in the 1·0–1·5 ml Yoshida foot sarcoma was higher than in resting muscle and normal skin (0·41 compared to 0·19 and 0·13 ml/g dry wt/min respectively) but was only 1/30th that of the kidney (12·51 ml/g/min).

After 1 h at 42°C, blood flow in normal rat skin increased from 0·13 to 2·33 ml/g/min and then decreased to 0·8–1·2 ml/g/min as heating continued to 2 and 3 h. In the tumour, 1 h at 42°C caused no alteration in blood flow, but continuation of the heating decreased blood flow to 0·15 ml/g/min at 2 h and to zero at 3 h.

The tumour differed from normal skin in its response to heat in 2 ways: (a) no increase in tumour blood flow occurred at raised temperature (b) heating of the tumour for longer than 1 h inhibited tumour blood flow. If applicable to tumours in general, these findings, with the concomitant increase in blood flow through normal tissues (*e.g.* skin) have important implications for heat therapy of cancer, since they enable the tumour-heating temperature to be reduced with time as well as protecting the normal tissues from heat damage.

### THE VALUE OF ROUTINE TESTS OF IMMUNOLOGICAL FUNCTION.

D. J. Hole, C. R. Gillis, B. R. Stack and E. Kirkwood, *Cancer Intelligence Unit, Ruchill Hospital, Glasgow.*

The potential value of using routine tests of immunological function sequentially as a means of assessing a patient's clinical condition has been assessed as part of a clinical trial examining the effect of immunotherapy in operable bronchial carcinoma. 70 patients were tested preoperatively at weekly intervals for 3 weeks, postoperatively 1 month after discharge and thereafter at 3-monthly intervals extending to 2 years; a total of 541 specimens. BCG and DNCB reactivity, lymphocyte counts and T- and B-cell determinations were made in addition to lymphocyte responses to PHA, pokeweed and PPD stimulation in each specimen. Apart from changes in BCG reactivity, which does relate to the patient's clinical progress, and a decline in T lymphocytes at about 9 months, there appears little consistency in the remainder of the tests. The fall in T lymphocytes does not occur early enough to act as an indicator for those about to exhibit recurrent disease. The remainder of the laboratory tests show some variations which may have relevance to the patients' progress, although this is not clear. It is considered that these results may be of significance for the future of immunological testing on a routine basis.

### LYMPHOBLASTOID CELLS AS INDICATORS OF LYMPHOKINE GENERATION.

E. Culbert and A. J. Cochran, *University Department of Pathology, Western Infirmary, Glasgow.*

We investigated the migration from capillary tubes of cells from human lymphoblastoid lines and normal human and mouse leucocytes. This is a metabolically active process, inhibited by low temperatures (4°C) and inhibitors of glycolysis, oxidative phosphorylation and RNA/protein synthesis. Migration is inhibited by medium conditioned by actively growing cultures, possibly a result of depletion of nutrients. The migration of normal human and mouse leucocytes and cells from the line QIMR-WIL was affected (enhanced or inhibited) by supernatants from cultures in which it was likely that lymphokines had been generated: PHA-stimulated lymphocytes and mixed-lymphocyte reactions (MLR). The migration of cells from the line NAMALWA was not affected by such supernatants, suggesting that these cells do not respond to migration-inhibitory lymphokines. Inhibitory activity in the supernatants of cultures of mouse spleen cells pulsed with PHA (1 μg/ml) increased with pulse duration and time of incubation after pulsing. MLR cultures also produced inhibitory activity which increased with time of incubation. Suitably controlled experiments indicated that the inhibitory activity was not due to a "medium-conditioning" effect.

Indicator lymphoblastoid cells are being assessed in a study of sensitization to tumour-associated antigens by co-culture of human lymphocytes and tumour or control cells.

### A RAPID METHOD OF SEPARATING LARGE AND SMALL THYMOCYTES FOR STUDY OF THEIR SURFACE PROPERTIES.

J. G. Salisbury, J. M. Graham and C. A. Pasternak, *Biochemistry Department, St George's Hospital Medical School, Tooting, London SW17.*

Thymocytes can be divided into 2 broad categories. The larger cells (diameter $>7$ μm) constitute 10–20% of the total and have been shown to be actively dividing. These cells give rise to small thymocytes ($\leq 7$ μm diameter) which constitute the majority of cells and are non-dividing (Metcalf & Wadriowski, 1966,

*Cancer Res.*, **26,** 483; Shortman & Jackson, 1974, *Cell. Immunol.*, **12,** 230). The aim of this study has been to separate these 2 populations and study their surface properties.

A simple method has been developed to separate the large and small cells from both rats and mice, involving the use of a new gradient material, Percoll (Pharmacia), which is a silica colloid. The cells are mixed with Percoll, the density of which is adjusted to lie between that of the large (1·07 g/ml) and small (1·086 g/ml) cells. At 1250 *g* the cells separate to give a band of large cells at the top and a pellet of small cells. A second spin ensures that the large-cell fraction is >85% pure. The separated populations of cells make a convenient system for the study of the surface properties of cycling and non-cycling cells.

Surface proteins of the 2 cell types are being studied using the lactoperoxidase-catalysed iodination ($I^{125}$) method (Hynes & Humphreys, 1974, *J. Cell Biol.*, **62,** 438). The proteins are extracted in SDS and separated by polyacrylamide gel electrophoresis.

**COMPARISON OF THE IMMUNOGENICITY OF CHEMICALLY INDUCED RAT AND MOUSE TUMOURS.** M. R. PRICE, R. G. DENNICK and L. W. LAW, *Cancer Research Campaign Labs., University of Nottingham, and Laboratory of Cell Biology, NCI, Bethesda, MD, U.S.A.*

Chemically induced rat hepatomas and sarcomas express individually distinct tumour-rejection antigens although immunization with acellular preparations of tumour antigens are ineffective in inducing protection against tumour-cell challenge. Immunization with radiation-attenuated hepatoma cells is optimally efficient when the cells retain a residual metabolic activity. Conversely, with the 3-methylcholanthrene-induced BALB/c murine sarcoma Meth A, resistance to challenge with $2 \times 10^4$ viable tumour cells is afforded by treatment with a single injection of $10^5$ X-irradiated cells and $\mu$g quantities of partially purified tumour antigen are immunoprotective (see, for example, Natori *et al.*, 1977, *Cancer Res.*, **37,** 3406). X-irradiated Meth A cells were shown to retain their immunogenicity even after glutaraldehyde treatment (0·001% to 0·1% for 30 min) mild heat treatment (45°C for 30 min) and 1mM iodoacetamide, each of these procedures being effective in abolishing the immunogenicity of rat hepatoma cells. These findings exemplify the powerful immunogenicity of the murine sarcoma in comparison to the rat tumours studied, and reflect the relative ease with which immunogenic preparations of acellular tumour antigens may be isolated from the Meth A sarcoma.

**HOST CELLS INFILTRATING TUMOURS *IN VIVO* AND *IN VITRO* REACTIVITY.** G. R. FLANNERY, R. A. ROBINS and R. W. BALDWIN, *Cancer Research Campaign Laboratories, University of Nottingham.*

Host lymphoid cells infiltrating a transplanted methylcholanthrene-induced rat sarcoma (Mc7) have been separated and tested for anti-tumour reactivity *in vivo* and *in vitro*. Cells derived from tumours growing progressively or from tumours regressing after BCG contact therapy were equally effective in the control of Mc7 *in vivo* when tested in Winn assays at effector: target cell ratios as low as 1·5:1. Cells derived from progressor tumours, when tested *in vitro* in 6h and 18h $^{51}$Cr-release and 60h $^{75}$Se-methionine-uptake microcytotoxicity assays, reacted with a range of other tumours, although the specificity of the reactivity varied with the different tests. Normal rat spleen cells, ineffective in the Winn assay, were also less effective than tumour-derived lymphocytes in the long-term cytotoxicity test, but showed significantly more reactivity in the short-term test, suggesting that the selenomethionine assay may be of greater value in predicting antitumour response *in vivo*.

**NATURAL IMMUNITY TO SOLID TUMOURS IN THE RAT IS MAINTAINED THROUGHOUT REPRODUCTIVE LIFE.** G. R. FLANNERY and C. G. BROOKS, *Cancer Research Campaign Laboratories, University of Nottingham.*

The spontaneous cytotoxicity of normal rat spleen cells towards a syngeneic methylcholanthrene-induced sarcoma (measured in a 6 h Cr-release assay) increased sharply in rats between 2 and 5 wks of age, but thereafter was maintained at a constant level until at least 20 months. Cell-mixing experiments indicated that the very low cytotoxicity in 2-wk-old animals was due to an absence of cytotoxic cells rather than the presence of suppressor cells. Removal of macrophages by carbonyl-Fe treatment caused no change in cytotoxicity levels at any age, indicating that only NK cell activity was being detected in this assay, and that cytotoxic or suppressor macrophages played no role. The persistence of NK-cell activity against sarcoma cells throughout reproductive life contrasts with the reported early decline of NK-cell activity against leukaemia target cells (Kiessling *et al.*, 1975, *Eur. J. Immunol.*, **5,** 117; Herberman *et al.*, 1975, *Int. J. Cancer*, **16,** 216; Nunn *et al.*, 1976, *J. Natl Cancer Inst.*, **56,** 393.

**SPONTANEOUS CYTOTOXIC ACTIVITY IN CELL SUSPENSIONS PREPARED FROM CONTROL AND HODGKIN'S DISEASE (HD) SPLEENS.** S. SERDENGEÇTI, D. B. JONES, S. V. DIXON and D. H. WRIGHT, *University Department of Pathology, General Hospital, Southampton.*

The paper reports the preliminary results of a study of spontaneous lymphocyte-mediated cytotoxicity (SLMC) against the human myeloid target line K562. In our system the main SLMC for normal blood is 65% ± 18 specific cytotoxicity at an effector:target ratio of 20:1. When compared at the same ratio control spleen tissue obtained incidental to abdominal surgery gave a mean of 19·15% ± 1·2 with an upper range of 21·3%. In 3 cases of HD the specific $^{51}$Cr-release values observed were 49·9%, 37·8% and 46·3%, suggesting that there is an increase in the SLMC-mediating population in the spleen in HD. Depletion of plastic adherent cells only slightly reduced the level of cytotoxicity and, where tested, the "B-enriched" cell population obtained by depletion of sheep-rosetting cells gave cytotoxicity equal to that of whole lymphocytes. The "T-enriched" cell fraction from both HD and control spleens gave only low $^{51}$Cr release, with a mean of 10%. These data add to those already published from this laboratory (Jones *et al.*, 1977, *Biomed. Express*, **27,** 177) indicating functional disturbances in the spleen in HD. The relationship of an increase in a cell population known to kill neoplastic targets preferentially (Jondal *et al.*, 1978, *Nature*, **272,** 62) to the development of the malignant process in HD is unclear.

**THE USE OF CELL FUSION TO INVESTIGATE THE ANTITUMOUR EFFECTS OF INTERFERON IN HUMAN NON-HODGKIN LYMPHOMA.** K. SIKORA, R. LEVY and T. MERIGAN, *Division of Oncology and Infectious Disease, Stanford University Medical Center, Stanford, California, U.S.A.*

Human leucocyte interferon was administered to 10 patients with lymphoid neoplasms. 5 patients with stage IV nodular lymphocytic poorly differentiated lymphoma demonstrated significant reductions in the size of previously stable or progressive abdominal and subcutaneous tumour masses. 4 of these patients had received no previous therapy. A similar course of interferon failed to influence the growth of advancing diffuse histiocytic lymphoma in 3 patients who had extensive prior radiotherapy and chemotherapy. 2 patients with chronic lymphocytic leukemia are currently receiving treatment.

The possible mechanisms by which interferon could be exerting its tumour-reducing effect were investigated. Detailed immunological monitoring of NLDP patients revealed an increase in the activity of circulating NK cells followed by increased responses of peripheral-blood lymphocytes to several mitogens and antigens. Attempts to obtain stable tumour lines *in vitro* from these patients failed. However, a set of human–mouse hybrid lines was constructed by fusing one patient's lymphoma cells with a mouse myeloma line. Several of the fusion products secreted human immunoglobulin of a single type. The effects of interferon on the kinetics of cell growth and immunoglobulin secretion were studied *in vitro* using these cells. Interferon is species-specific and as expected it has no effect on the mouse myeloma line. Growth retardation was noted in some of the hybrids and an attempt was made to map this effect to a specific chromosome.

**SERUM PROTEIN CHANGES IN BREAST CANCER.** J. W. HILLYARD, J. W. KEYSER, R. G. NEWCOMBE and D. J. T. WEBSTER, *University Departments of Surgery, Medical Biochemistry and Medical Statistics, Welsh National School of Medicine, Cardiff.*

Current methods of staging breast cancer are insensitive, and unfortunately no specific tumour marker has been detected in this disease. However, nonspecific protein changes are known to occur and the data from a small preliminary series suggest that some are related to stage (Coombes, *et al.*, 1977, *Lancet*, i, 132).

We have studied prospectively 168 patients suspected of having breast cancer: 39 were found to have benign breast disease and 129 breast cancer of the following Manchester stages: I: 42, II: 28, III: 44, IV: 15. 14 serum proteins were measured before treatment: albumin, $\alpha_1$ antitrypsin, prealbumin, $\alpha_2$ macroglobulin, $\alpha_1$ acid glycoprotein, caeruloplasmin, immunoglobulins G, A and M, transferrin, ferritin, C-reactive protein, pregnancy-associated macroglobulin and $\beta_2$ microglobulin.

There were statistically significant differences in both of the comparisons, benign: malignant, and Stage I: IV in the case of 3 proteins ($\alpha_1$ acid glycoprotein, C-reactive protein and $\beta_2$ microglobulin).

3 of the other proteins (ferritin, $\alpha_1$ antitrypsin and prealbumin) showed significant differences between Stage I and IV, but not between benign and malignant.

There was so much overlap between stages for all 6 of these proteins that none of them would aid initial management if used individually.

**ADJUVANT CHEMOTHERAPY IN EARLY BREAST CANCER.** D. C. SMITH, D. J. ROSS, A. R. RUSSELL and C. S. MCARDLE, *Departments of Surgery and Radiotherapy, Victoria Infirmary, Glasgow.*

Although adjuvant chemotherapy significantly improves disease-free survival after radical surgery for primary breast cancer when the axillary nodes are involved (Bonnadonna *et al.*, 1976, *N. Engl. J. Med.*, **294**, 405), its value after less radical surgery is not known. In this study, initiated in 1976, patients with early breast cancer were treated by simple mastectomy and axillary clearance. If the axillary nodes were involved, patients were randomized to receive postoperative radiotherapy, chemotherapy (CMF) or radiotherapy followed by chemotherapy. 58 patients have now been followed up for between 1 and 2½ years.

Of 24 women receiving radiotherapy alone, 5 died of breast cancer within 2 years and 3 others had developed widespread metastases (Table). In contrast, 2/18 patients receiving chemotherapy alone had died, and only 2/16 patients receiving both modalities of treatment had died.

These early results suggest that, after simple mastectomy and axillary clearance, adjuvant chemotherapy improves survival in patients with early breast cancer.

| Treatment | Recurrence: Local | Recurrence: Disseminated | Dead |
|---|---|---|---|
| Radiotherapy (24) | 1 | 3 | 5 |
| Radiotherapy + Chemotherapy (16) | 1 | – | 2 |
| Chemotherapy (18) | 1 | – | 2 |

**PSYCHIATRIC MORBIDITY AFTER CHEMOTHERAPY AND RADIOTHERAPY FOR BREAST CANCER.** A. F. COOPER, A. V. M. HUGHSON, C. S. MCARDLE, A. R. RUSSELL and D. C. SMITH, *Depts. of Psychiatry and Surgery, Victoria Infirmary, Glasgow.*

Adjuvant chemotherapy following mastectomy may prolong disease-free survival, but there is little information on its quality (British Breast Group, 1976, *Br. Med. J.*, ii, 861). Following simple mastectomy, psychiatric morbidity was studied during a trial of adjuvant chemotherapy, patients being randomly allocated to chemotherapy (C), radiotherapy plus chemotherapy (RT&C) or radiotherapy (RT). Two self-rating scales of morbidity were used: the General Health Questionnaire (GHQ) (Goldberg, 1972; OUP) providing a non-specific measure; and the Leeds Self-assessment of Depression (LSM) and Anxiety (LSA) Scales (Snaith *et al.*, 1976, *Br. J. Psychiat.*, **128,** 156). Two groups of survivors were compared: Group I (C and RT&C) and Group II (RT).

Mean scores on the LSD (Table) show that depression is significantly greater in Group I than in Group II at both 12 and 18 months after mastectomy. On the GHQ, psychiatric morbidity was significantly greater at 12 months, and on the LSA anxiety was significantly greater at 18 months. Results indicate that psychiatric illness, particularly depression, is greater in patients completing a one-year course of chemotherapy than in those who had radiotherapy one year before: and that differences between the groups are still evident 6 months later.

TABLE.—*Mean scores on LSD at* 12 *and* 18 *months after mastectomy*

| After mastectomy | Group I Mean ± s.e. | No. | Group II Mean ± s.e. | No. | *P* |
|---|---|---|---|---|---|
| 12 months | 4·8 ± 0·8 | 29 | 1·9 ± 0·6 | 16 | < 0·01 |
| 18 months | 3·6 ± 0·9 | 20 | 1·2 ± 0·3 | 12 | < 0·02 |

**THE MANAGEMENT OF MALIGNANT EFFUSIONS WITH BLEOMYCIN.** J. M. TROTTER, J. F. B. STUART, F. MCBETH, J. G. MCVIE and K. C. CALMAN, *Department of Clinical Oncology, University of Glasgow.*

Intracavitary bleomycin was used in a total of 23 patients with malignant effusions, with an overall complete and partial response rate of 73% in 22 evaluable patients. 78% of intrapleural and 69% of intraperitoneal instillations of bleomycin produced significant objective and subjective responses. There were 2 possible drug-related deaths with this therapy, which was otherwise well tolerated with low toxicity. Lower average doses than those previously described (Paladine *et al.*, 1976, *Cancer*, **38,** 1903) have proved as effective in controlling effusions. In addition, control of pleural effusions was accomplished without the need for under-water sealed drainage. Evaluation of the response of different malignancies was statistically not possible, owing to small numbers. High serum levels of bleomycin in one of the treatment-related deaths suggests that intracavitary doses of bleomycin in excess of 40 mg/m² should be avoided in the elderly.

**A PROSPECTIVE STUDY OF ANTI-EMETIC DRUGS IN THE MANAGEMENT OF NAUSEA AND VOMITING ASSOCIATED WITH CYTOTOXIC THERAPY.** C. MORRAN, D. A. ANDERSON, D. C. SMITH and C. S. MCARDLE, *Department of Surgery, Victoria Infirmary, Glasgow.*

Toxic side-effects, particularly nausea and vomiting, limit the usefulness of some cytotoxic regimens. The value of anti-emetic drugs in controlling these symptoms is unproved. In a prospective randomized study of anti-emetic drugs, active agents were compared with placebo in women receiving adjuvant chemotherapy (cyclophosphamide, methotrexate and 5-fluouracil) for breast cancer.

83% of those receiving placebo developed nausea and 78% vomited, the peak incidence being between 12 and 36 h after injection. Severe nausea and vomiting occurred in 65% and 52% of assessments respectively. Metoclopramide, cyclizine and fluphenazine failed to alter the incidence or severity of gastrointestinal upset. The combination of fluphenazine and nortriptyline significantly reduced the severity and duration of nausea and vomiting (Table). These data suggest that the combination of a phenothiazine and a tricyclic antidepressant may be effective in controlling nausea and vomiting associated with this form of chemotherapy.

| Affected with: | Placebo (%) | Fluphenazine/ Nortriptyline (%) | *P* |
|---|---|---|---|
| Nausea | 83 | 53 | NS |
| Severe nausea | 65 | 21 | < 0·01 |
| Vomiting | 78 | 47 | NS |
| Severe vomiting | 52 | 11 | < 0·01 |
| Days free of nausea | 41 | 74 | < 0·0005 |
| Days free of vomiting | 74 | 90 | < 0·005 |

### CYCLOPHOSPHAMIDE-INDUCED CYSTITIS: ITS CAUSE AND POSSIBLE CLINICAL SIGNIFICANCE.

P. J. Cox and G. Abel, *Department of Biochemical Pharmacology, Chester Beatty Research Institute, London SW3.*

Cyclophosphamide {2-[bis(2-chloroethyl)-amino]tetrahydro - 2H - 1, 3, 2 - oxaza - phosphorine 2-oxide} is widely used as an anti-tumour drug and as an immunosuppressive agent in non-neoplastic conditions. This compound and its isomer ifosfamide are the only clinically used alkylating agents which cause specific urological damage, namely interstitial haemorrhagic cystitis. This phenomenon was studied in the rat, using an acute technique (Levy & Harris, 1977, *Biochem. Pharmacol.*, **26,** 1015), and optimum conditions for protection from toxicity by *N*-acetyl-L-cysteine were found. Various metabolites and analogues of cyclophosphamide were tested, including phosphoramide mustard, nornitrogen mustard, 5,5-dimethylcyclophosphamide, diethylamine, diethyl-cyclophosphamide [2(diethylamino)tetrahydro-2H-1,3,2-oxazophosphorine 2-oxide], acrolein and 3-hydroxypropylmercapturic acid. Only those capable of releasing acrolein in the bladder caused oedema and haemorrhage (Cox, 1979, *Biochem. Pharmacol.*, in press). Protection from toxicity with *N*-acetyl-L-cysteine was possible in all cases.

An apparently high proportion of tumours secondary to cyclophosphamide treatment is found in the bladder (Puri & Campbell, 1977, *Lancet*, i, 1306). This may be the consequence of 2 occurrences: the presence in patients' urine of strongly alkylating metabolites of cyclophosphamide, and the rapid hyperplastic response of the bladder epithelium to damage by acrolein. Thus patients who may have subclinical cystitis may be at as much risk of drug-induced malignant changes as patients suffering severe bladder toxicity.

### TESTOSTERONE METABOLISM BY HOMOGENATES AND CULTURED EXPLANTS OF HUMAN BREAST CANCER.

W. R. Miller, F. W. Brannan and A. P. M. Forrest, *Department of Clinical Surgery, Royal Infirmary, Edinburgh.*

Homogenates of human breast cancer metabolize testosterone to Δ4 androstenedione, 5α reduced metabolites and oestrogen (Miller & Forrest, 1976, *Br. J. Cancer*, **33,** 116; Li *et al.*, 1976, *Steroids*, **28,** 561). However, less is known about the ability of explants of human breast cancers maintained in culture to metabolize steroid hormones. The transformation of $^{3}$H-testosterone by homogenates of 11 human breast cancers has been compared with that by explants of the same tumours in organ culture. Both homogenates and cultures of all tumours metabolize testosterone to Δ4 androstenedione, 5α dihydrotestosterone, 5α androstanedione and 5α androstanediols (3α 17β and 3β 17β). However, whereas oestradiol synthesis was detected in 4 homogenates, evidence for oestrogen production was obtained in only one set of cultures. Metabolism was quantitatively similar in both homogenates and cultures, except for the production of Δ4 androstanedione, which was greater in cultured systems. Tumours from 3 patients showed higher 5α reductase activity than the remaining tumours, which was evident both in homogenate and cultured systems. In 7 cultures metabolites were characterized separately in explants and culture medium. The percentage production of metabolites was always higher in explants compared media, whereas the proportion of testosterone unmetabolized was correspondingly lower. In summary it has been demonstrated that both homogenates and explants of human breast cancer maintained in culture have similar potential to convert testosterone to other C19 steroids, but perhaps not to oestrogen.

### THE EFFECT OF PROLACTIN AND OTHER HORMONES ON LACTALBUMIN PRODUCTION BY NORMAL AND NEOPLASTIC HUMAN BREAST TISSUE.

G. D. Wilson, K. L. Woods* and A. Howell, *Departments of Medicine and *Clinical Pharmacology, University of Birmingham.*

We have previously demonstrated that about 40% of human breast tumours contain measurable amounts of lactalbumin (Woods *et al.*, 1977, *Lancet*, ii, 14). In order to investigate its utility as a tumour marker, a better understanding of lactalbumin physiology in normal and neoplastic tissue is required.

Human breast tissue was maintained in organ culture, in serum-free media, for up to 10 days. Viability was assessed by light microscopy, and lactalbumin in the surrounding medium was assayed by radioimmunoassay (Woods & Heath, 1977, *Clin. Chim. Acta*, **78,** 129). Hormones were added to medium in the following concentrations, 2–50 $\mu$g/ml ovine prolactin, 10 $\mu$g/ml bovine insulin, 1 ng/ml oestradiol, 1 $\mu$g/ml progesterone, 1 $\mu$g/ml hydrocortisone.

Of 8 premenopausal normal breast samples tested, 5 have produced lactalbumin in culture. All 5 responded to prolactin treatment over 4 days, one of them producing lactalbumin when there was none detectable initially. One biopsy sample of pregnant breast tested also responded to prolactin treatment, producing 200× more lactalbumin in culture than normal breast. 2/4 postmenopausal breast biopsy samples produced lactalbumin and one increased synthesis and release of lactalbumin after prolonged exposure to prolactin. One fibroadenoma has been tested which responded to prolactin. Of 9 scirrhous carcinomas, 5 produced lactalbumin and none responded to prolactin. A dose-related response to prolactin was found with normal premenopausal breast tissue but not with tumour tissue from the same patient.

Of other hormones tested only insulin and the combination of insulin, prolactin and oestrogen have had an effect on normal tissue. No hormone or combination of hormones has stimulated lactalbumin production in malignant tissue.

In summary, lactalbumin production can be stimulated in normal, but not in malignant breast tissue. Malignant tissue may have defective prolactin receptors or none.

### THE POSSIBLE RELATIONSHIP BETWEEN SEX CHROMATIN AND OESTRADIOL RECEPTORS IN HUMAN BREAST CANCER.

N. P. Bishun, M. Smethurst and D. C. Williams, Research Department, *The Marie Curie Memorial Foundation, Oxted, Surrey.*

Sex chromatin (Barr body) frequency and cytoplasmic oestradiol receptors have been determined in 46 human breast tumours. The division into sex chromatin (SC) positive and negative groups was made at frequencies of 11%, 16% and 21%. The proportion of tumours with oestradiol receptors in the SC positive and negative groups varied with the percentage frequency taken for this division. At the 11% level there were too few tumours in the SC negative group to form any definite conclusions, but when making the division at 16% we found that the tumours with the higher frequency of SC were more likely to have oestradiol receptors. At the 21% level we found that SC frequency was independent of oestradiol receptors.

### ELASTOSIS AND ENDOCRINE STATUS IN HUMAN BREAST CANCER.

J. R. W. Masters*, R. R. Millis†, R. J. B. King‡ and R. D. Rubens§, **Institute of Urology, St Paul's Hospital, London WC2, †§Imperial Cancer Research Fund, Breast Cancer Unit, Guy's Hospital, London SE1, and ‡Imperial Cancer Research Fund, Lincoln's Inn Fields. London WC2.*

Subjective histological assessments in primary human breast tumours have shown that elastosis is related to prognosis (Shivas & Douglas, 1972, *J. R. Coll. Surg.* (*Edin.*), **17,** 315), oestrogen-receptor status (Masters *et al.*, 1976, *Br. J. Cancer*, **33,** 342) and menstrual status (Masters *et al.*, 1978, *Eur. J. Cancer*, **14,** 303). Further studies have shown a relationship between elastosis in the primary tumour and the response to endocrine therapy (measured objectively using UICC criteria) of advanced breast cancer, as shown in the Table. While only 6/51 tumours contained extensive elastosis, 3 of these patients obtained a complete response ($P < 0{\cdot}005$). In addition, a combination of elastosis and oestrogen receptor status provided a better predictive index than either feature alone. In conclusion, elastosis assessment is simple, requires neither additional tissue nor expertise, can readily be included in routine pathology reports or measured retrospectively, and may be of value in the management of breast cancer.

| | Elastosis category | | |
|---|---|---|---|
| Response to therapy | 0 (No elastosis) | 1 (Elastosis present) | 2 (Gross elastosis) |
| Complete | 0 | 3 | 3 |
| Partial | 2 | 5 | 0 |
| No change | 3 | 5 | 1 |
| Progressive disease | 9 | 18 | 2 |
| Total | 14 | 31 | 6 |

**OESTROGEN RECEPTORS: A GUIDE TO PROGNOSIS IN EARLY BREAST CANCER.** T. Cooke, P. Maynard, D. George, K. Griffiths and R. Shields, *Department of Surgery, University of Liverpool, and Tenovus Institute for Cancer Research, Cardiff.* (Introduced by M. Moore.)

The aim of this study was to define the role of oestrogen receptor (ER) analysis in primary breast cancer.

ER was measured by the dextran-coated charcoal technique in 286 women undergoing mastectomy for early breast cancer. These patients have been followed up for 3–39 (mean 19) months.

Data were analysed by life tables and the log rank test.

64 women have developed proven recurrent disease.

Patients with $ER^+$ tumours had significantly fewer recurrences ($P < 0{\cdot}001$) than those who were $ER^-$.

On further analysis there emerged an interesting relationship between ER and the histology of the lymph nodes. In lymphnode-negative patients there was a significantly higher rate of recurrence ($P < 0{\cdot}01$) for $ER^-$ tumours than for $ER^+$ ones. In these patients ($LN^-$ $ER^-$) the rate of recurrence was the same as in all patients with involved nodes.

Patients with involved nodes and $ER^-$ tumours had the highest rate of recurrence, significantly above that of all other groups.

We conclude that ER is a prognostic guide in early breast cancer and that it is independent of lymphnode status. We have defined a subgroup of patients ($LN^-$ $ER^-$) who, despite the absence of axillary-node spread, stand the same high risk of early recurrence as patients with nodal involvement.

**THE PROGNOSTIC SIGNIFICANCE OF NUCLEAR OESTROGEN RECEPTORS IN BIOPSY SPECIMENS OF PRIMARY BREAST CANCERS.** D. C. Smith, D. Crawford, L. Laing and R. E. Leake, *Division of Surgery, Victoria Infirmary, and University Department of Biochemistry, Glasgow.*

The presence of cytoplasmic oestrogen receptor (ER) in biopsy specimens of metastatic breast cancer is now known to improve the accuracy of prediction of response to hormonal manipulation. Knight *et al.* (*Cancer Res.*, 1977, **37,** 4669) have reported that the absence of cytoplasmic ER in primary breast cancers is associated with early recurrence, independent of other known prognostic factors. In this study, ER was assayed in both the cytoplasmic (C) and nuclear (N) fractions of biopsy samples of primary breast cancers. The results in 109 patients were correlated with survival. 4 groups were defined according to the presence or absence of receptor in both cellular fractions—$C^+/N^+$ (26), $C^-/N^-$ (59), $C^-/N^+$ (6) and $C^+/N^-$ (18). Follow up ranged from 13 to 28 months. Disease-free survival was highest in the $C^+/N^+$ group (77%), and poorest in the $C^+/N^-$ group (50%). Of the 77 patients with no detectable nuclear receptor, 26% had died of their disease, compared to only 9% of those whose biopsy specimens contained nuclear receptor. These results suggest that the measurement of nuclear ER adds to the prognostic information achieved by measuring cytoplasmic ER alone.

**SIMULTANEOUS ESTIMATION OF CYTOPLASMIC OESTROGEN AND PROGESTERONE AND NUCLEAR OESTROGEN RECEPTORS IN HUMAN BREAST TUMOURS AND CORRELATION WITH CLINICAL RESPONSE.** L. G. Skinner, D. M. Barnes and G. G. Ribeiro, *Christie Hospital and Holt Radium Institute, Manchester.*

Of 336 primary and 148 metastatic human breast tumours examined, 58% and 43% respectively were found to have positive cytoplasmic oestradiol-receptor ($ER_c$) activity. 59% of primary and 56% of metastatic tumours with $ER_c$ activity were also found to possess cytoplasmic progestin-receptor ($PR_c$) sites. 13% of $ER_c^-$ tumours were $PR_c^+$.

Of 74 patients with advanced metastatic breast cancer, 57% of those with $RE_c^+$ tumours had a clinical response (complete or partial) to endocrine therapy. Where the tumour was assayed for both $ER_c$ and $RP_c$ activity before hormone therapy was started, 9/12 (75%) of patients whose tumour contained both $ER_c$ and $PR_c$ sites responded, whereas only 3/30 (10%) of patients with negative $ER_c$ and $PR_c$ had a clinical response, an indication that tumours containing both receptors are most likely to show a high response rate to endocrine therapy.

Nuclear oestrogen receptor ($ER_n$) assay, carried out on pellets from tumour-tissue homogenates, has recently been studied in an attempt to improve further the prognostic value of receptor measurements. Of 284 tumours assayed, 77 were positive for all 3 receptors, 130 lacking in any receptor activity. Of the limited number of patients who have become eligible for assessment to date, 5/6 with tumours positive for all 3 receptors have shown a clinical response, whereas 0/9 patients with negative status have responded.

Patients with $ER_c^+$ and $PR_c^+$ primary tumours tended to have a longer disease-free interval than patients with $PR_c^-$ tumours, irrespective of whether these were $ER_c^+$ or $ER_c^-$.

### PHYSICAL PROPERTIES OF OESTROGEN RECEPTORS FROM BREAST CANCER BIOPSY SPECIMENS IN RELATION TO CLINICAL PREDICTIONS.

L. LAING and R. LEAKE, *Department of Biochemistry, University of Glasgow.*

Oestrogen-receptor status has, in recent years, become a useful guide to potential response to hormone-based therapy for breast cancer. It may also serve as a good prognostic index. We have previously shown that detection of nuclear oestrogen receptors in breast-cancer biopsy specimens may give additional, valuable clinical information (Laing *et al.*, 1977, *Lancet*, ii, 168). An understanding of the full significance of these nuclear receptors depends on a knowledge of both their exact cellular localization and their functional integrity. Potential abnormalities in the translocation to the nucleus of oestrogen receptor have been investigated in certain breast tumours. The off-rate of steroid (the rate at which steroid dissociates from its receptor) has been measured at various temperatures for hormone-receptor complex from both cytoplasmic and nuclear fractions. It was found to be significant even at 0°C. The distribution of hormone-receptor complex between the various subcellular fractions has been determined and is considered in relation to potential response to hormone therapy. The value, in terms of stabilizing steroid receptor, of different protease inhibitors (Trasylol and PMSF), both independently and in combination, has been determined in relation to development of a routine procedure for receptor assay. An assay procedure based on our conclusions from the above observations will be presented in detail.

### OESTROGEN RECEPTORS AND RESPONSE TO TAMOXIFEN IN SOME TUMOURS OTHER THAN BREAST.

F. R. MACBETH, K. C. CALMAN, L. LAING and R. E. LEAKE, *Departments of Oncology and Biochemistry, Glasgow University.*

There have been anecdotal reports of response of tumours other than breast to the anti-oestrogen tamoxifen. We are investigating this further on a clinical basis, and also by assaying cytoplasmic and nuclear fractions of tumour biopsy specimens for oestrogen receptor. If oestrogen receptor is present, and there have been reports of it being detected in malignant melanoma, and a number of normal tissues including kidney, this would provide a scientific basis for hormonal therapy in such tumours.

No significant cytoplasmic or nuclear oestrogen receptor has been found in 5 biopsy specimens of malignant melanoma, 2 of carcinoma of stomach, 2 of renal cell carcinoma, nor in the adjacent normal kidney tissue from one specimen. 44 samples of rectal and colonic carcinoma have been assayed. Cytoplasmic receptor was detected in 2 and both nuclear and cytoplasmic receptor in a third sample.

A number of patients with advanced disease, who were failing on conventional therapy, have been treated with tamoxifen. There have been no responses in 6 patients with malignant melanoma, 3 with carcinoma of colon, 1 with carcinoma of oesophagus and 1 with adenocarcinoma of stomach. There is therefore at present no clinical evidence nor scientific basis for treating these tumours with anti-oestrogen therapy, although the study is continuing.

### HORMONE RECEPTORS IN MALIGNANT MELANOMA.

P. RÜMKE, C. B. KORSTEN and J. P. PERSIJN, *Department of Internal Medicine and Department of Clinical Chemistry, Division of Endocrinology, The Netherlands Cancer Institute, Amsterdam.*

The aim of the study is the detection of oestrogen and/or androgen receptors in the cytosol of metastases of malignant melanoma, in the hope that successful hormonal therapy in advanced disease may be related to their presence. Oestradiol ($E_2$) and dihydrotestosterone (DHT) receptors were assayed in 43 histologically proven skin and lymphnode metastases from 34 (17♂ and 17♀) melanoma patients. Only in 2 (female) patients did the metastases contain more than 10 fmol $E_2$ receptor/mg cytosol protein, which according to standards used in breast cancer could indicate a hormonal dependency. In 4 other patients (3♂ and 1♀) DHT receptor levels were more than 10 fmol/mg cytosol protein. In 2 young male patients with advanced disease with DHT levels of 13 and 16, 7 fmol/mg cytosol, hormonal treatment was unsuccessful.

### MELPHALAN AND URINARY OESTROGENS.

J. MAXWELL ANDERSON*, J. K. GRANT* and G. GETTINBY†, **Royal Infirmary, Glasgow, and †University of Strathclyde, Glasgow.*

A test of the concept of chemical suppression of ovarian function by cytotoxic drugs previously reported (Anderson *et al.*, 1978, *Br. J. Cancer*, **37**, 477) revealed no supportive evidence from follicular and luteal-phase measurements of plasma oestradiol ($E_2$) in 6 premenopausal women prescribed repeated 5-day courses of melphalan after mastectomy for cancer. To circumvent the difficulties of frequent repeated blood sampling, 3 of these patients were studied in greater depth. Early-morning urine samples were collected on alternate days for 6 months. 5-day courses of oral melphalan were taken, to cover the expected midcycle peaks of $E_2$, in alternate months. No differences in excretion of total oestrogens (E) between the melphalan and non-melphalan cycles were observed, failing to support the concept of ovarian suppression. Unexpected early peaks of E were consistently noted in most cycles. 12 normal premenopausal women were examined similarly and their cycles showed no evidence of the early oestrogen peaks seen in the melphalan-treated patients. This apparent anomaly is being examined further, in comparison with the urinary oestrogen profiles of regularly menstruating cancer bearers never prescribed chemotherapy.

**IMPLICATIONS OF GOMPERTZ GROWTH KINETICS FOR THE CHOICE OF TREATMENT SCHEDULES IN CANCER THERAPY.** T. E. WHELDON and G. F. BRUNTON, *Radiobiology Research Group, Glasgow Institute of Radiotherapeutics and Oncology, Belvidere Hospital, Glasgow.*

Most experimental tumours grow more rapidly when small than large but, as regenerating normal cell populations do likewise, this provides no basis for treatment scheduling to discriminate between normal and malignant populations, at least for the great majority of cytotoxic agents. This view is contrary to that of Norton and Simon (1977 *Cancer Treat. Reps.*, **61,** 1307), who have advocated unorthodox scheduling for cytotoxic agents in general, on the basis of tumour growth kinetics.

However, there may exist a small minority of cytotoxic agents whose limiting host toxicity is cumulative with dose, but largely independent of the treatment schedule by which it is given. Examples of this may include the limiting cardiotoxicity of adriamycin and the limiting pulmonary toxicity of bleomycin.

For these agents in particular, mathematical-model studies suggest that tumours following Gompertz growth kinetics, over at least the upper part of the growth range, may be treated more effectively by "late-intensity" schedules than by schedules of more conventional design.

Experimental appraisal of "late-intensity" scheduling seems warranted for the special case of those agents whose limiting toxicity is cumulative with dose.

**THE MODE OF ACTION OF "CHIP" STUDIED BY THE ANALYSIS OF CHROMOSOME-ABERRATION PRODUCTION.** E. BOCIAN*, M. LAVERICK and A. H. W. NIAS, *The Richard Dimbleby Department of Cancer Research, St Thomas's Hospital Medical School, London SE1.*

* Present address: Zaktad Radiobiologii i Ochrony Zdrowia, Instytut Badan Jadrowych, Dorodna 16, 03–195 Warszawa, Poland.

Platinum complexes form inter- and intrastrand crosslinks in DNA, and in this they are similar in their action to bifunctional alkylating agents (Roberts & Pascoe, 1972, *Nature*, **235,** 282). According to the classification of Bender (1974, *Mut. Res.*, **23,** 197) these agents fall into the 3rd class of chemicals which produce lesions of the "delayed type" and which can be repaired by recombination and post-replication processes. Cis-dichloro bis(cyclopentylamine) platinum II (PAD) has been shown to fall into this 3rd class of chemicals with respect to its chromosome-aberration production (Szumiel & Nias, 1976, *Chem-Biol. Interact.*, **14,** 217) in Chinese hamster ovary (CHO) cells. We now report results in the same cell system with cis-dichloro-bis (isopropylamine) trans-dihydroxy platinum IV (CHIP). Only chromatid-type aberrations were seen but all types were produced at the 1st mitosis after treatment in $G_1$ and early S phases. This is contrary to the findings with PAD, where exchanges were only seen at the 2nd mitosis after treatment. The majority of aberrations were breaks and gaps and the frequency of aberrations was dose-dependent. No aberrations were seen at the 1st mitosis after treatment of cells in $G_2$. This confirms the hypothesis that lesions produced by CHIP can only be seen after DNA synthesis has taken place on the damaged template.

(Supported by Grant Number CA 20593 from the U.S. National Cancer Institute.)

**ACTINOMYCIN-D PERTURBATION OF NUCLEIC ACID SYNTHESIS IN SYNCHRONIZED MOUSE TUMOUR CELLS *IN VITRO*.** J. V. WATSON and S. H. CHAMBERS, *MRC Clinical Oncology Unit, The Medical School, Hills Road, Cambridge.*

Flow-cytometry systems enable RNA and DNA to be estimated simultaneously in intact single cells by measuring the respective red and green fluorescence emissions after acridine-orange staining (Watson & Chambers, *Cell Tissue Kinet.*, 1978, **11,** 415). These techniques have been used to study the alterations in nucleic acid content of synchronized EMT6/M/CC cells due to low doses of actimomycin-D (ACT-D). The concentrations used were 10 and 100 ng/ml as these span the therapeutic range that can be achieved in man. With continuous exposure to the lower dose of ACT-D from mid S-phase, early S and mid $G_1$ it was found that: (1) the RNA remained constant at the level attained when exposure began, (2) a progressive delay in the cycle time occurred with increasing exposure time, (3) mitosis was not inhibited. At the higher dose level we found that: (1) the RNA decreased approximately exponentially from the level when exposure began, (2) continuous exposure from the end of $G_1$ did not inhibit mitosis although the RNA level was decreasing, but the cycle time was increased. Continuous exposure from the beginning of the cycle at both dose levels inhibited mitosis. A number of explanations can be given for these results, but the possibility that RNA messenger which is responsible for subsequent mitosis is synthesized during $G_1$ and not during a late RNA synthesis should be considered in this cell line.

**THE RESISTANCE OF HYPOXIC MAMMALIAN CELLS TO CHEMOTHERAPEUTIC AGENTS.** E. SMITH, I. J. STRATFORD and G. E. ADAMS, *Physics Division, Institute of Cancer Research, Belmont, Sutton, Surrey.*

It is now unequivocably proven that hypoxic cells are one of the limiting factors in the local control of some tumours by radiotherapy. The importance of these *potentially clonogenic* hypoxic cells has apparently received scant attention in the treatment of solid tumours with chemotherapy. Recently, it was shown that bleomycin was less toxic towards exponentially growing cells which had been rendered hypoxic than to fully aerobic cells (Roizin-Towle & Hall, 1978, *Br. J. Cancer*, **37**, 254). We have confirmed this result, and in addition have shown that hypoxic cells are more resistant to treatment by actinomycin D, adriamycin, 5-FU, Ara-C and vincristine.

In particular, the toxicity of adriamycin towards hypoxic cells is dependent upon the length of time for which the cells were previously rendered hypoxic at 37°C. Results will also be presented which show that previously hypoxic cells do not immediately recover their sensitivity to adriamycin when they are made aerobic again.

**METHOTREXATE PROTEIN BINDING.** W. H. STEELE, J. F. B. STUART, J. R. LAWRENCE and K. C. CALMAN, *University Department of Materia Medica and Clinical Oncology, University of Glasgow.*

Published estimates for the protein-binding of methotrexate (MTX) range from 60 to 94%. We have assessed the protein-binding of MTX in serum from 8 healthy subjects by continuous ultrafiltration; drug levels were measured by radioimmunoassay.

MTX was found to bind predominantly to serum albumin. Over the range 1–30 $\mu$M the binding was linear at $95 \cdot 1 \pm 2 \cdot 3\%$ (mean ± s.d.) but at concentrations greater than 50 $\mu$M deviation from linear binding was found. Protein-binding parameters were obtained from the analysis of Scatchard curves, which indicated 2 distinct groups of binding sites. In the high-affinity group these were $0 \cdot 16 \pm 0 \cdot 05$ binding sites ($N_1$) with an intrinsic association constant of $71 \cdot 15 \times 10^4/\text{M} \pm 35 \cdot 98$ ($K_1$), whereas in the lower affinity group $N_2 = 2 \cdot 01 \pm 0 \cdot 93$ and $K_2 = 0 \cdot 18 \times 10^4/\text{M} \pm 0 \cdot 15$. Protein-binding of a cytoxic agent could be of critical importance, in that anomalies therein might greatly affect the distribution and excretion of the agent. This is particularly important where protein-binding is saturable within a concentration range commonly produced *in vivo*, as we have demonstrated for MTX. Further, the presence of neoplastic disease, response or failure of response, and nutritional status could affect the binding parameters, thereby altering the disposition characteristics of MTX.

**PHARMACOKINETICS OF BLEOMYCIN FOLLOWING I.M., I.V. OR INTRACAVITARY ADMINISTRATION.** J. F. B. STUART, W. AHERNE, L. PRASAD, S. JAMES, J. M. TROTTER and J. G. MCVIE. *Department of Clinical Oncology, University of Glasgow and Department of Biochemistry, University of Surrey.*

The major limitation of bleomycin administration by the i.v. or i.m. routes is the occurrence of pulmonary fibrosis. This side effect, usually irreversible, is increasingly common above a total cumulative dose of 300–250 mg/m². Bleomycin has been advocated in high dose for treatment of malignant pleural and peritoneal effusions. Alberts (1978) *Proc. Am. Ass. Cancer Res.*, 305, have suggested that 40% of an intrapleural dose is absorbed, thus contributing to the total dose limitation. A group of 5 levels have been studied in an attempt to reproduce these data. Bleomycin levels were measured in plasma samples removed at 11 times after injection of the drug either i.m., i.v. or i.p. (intrapleural). Close agreement with Alberts's data has been obtained, *viz*:

| | i.m | i.v. | i.p. |
|---|---|---|---|
| $T_{\frac{1}{2}}$ (β) (h) | 4·7 | 4·3 | 6·3 |
| AUC (μg min/ml) | 864 | 903 | 388 |

Thus exposure of the systemic circulation to bleomycin after 30 mg intrapleural injection is half of that after an i.m. or i.v. dose.

**EFFECT OF PHENOBARBITONE AND PHENYTOIN ON THE PHARMACOKINETICS AND TOXICITY OF MISONIDAZOLE.** P. WORKMAN, C. R. WILTSHIRE and N. M. BLEEHEN, *MRC Clinical Oncology Unit, Hills Road, Cambridge.*

Concentrations of the hypoxic cell radiosensitizer misonidazole (MIS, Ro 07-0582) and its O-demethylated metabolite Ro 05-9963 were determined in plasma (or blood), tumour and brain after an injection of 1 g/kg MIS i.p. to mice pretreated with phenobarbitone, phenytoin or saline vehicle. Analysis was HPLC (Workman *et al.*, 1978, *J. Chromatogr.* **145,** 507).

Phenobarbitone and phenytoin pretreatment did not alter the peak MIS concentration in plasma, brain or tumour, but did reduce the apparent elimination half-life ($t\frac{1}{2}$) and area under the curve (AUC) for MIS in all 3. The decrease in $t\frac{1}{2}$ from ~2 to 1 h was associated with an increased Ro 05-9963 metabolite concentration. The AUC for total 2-nitroimidazole was also reduced in plasma, tumour and brain by 20–50%.

Both phenobarbitone and phenytoin increased the acute $LD_{50}$ for MIS in mice from 1·54 g/kg to 1·90 g/kg and 1·78 g/kg respectively ($P <$ 0·001).

Preliminary studies in man indicate that phenytoin reduces the MIS $t\frac{1}{2}$ by increasing MIS O-demethylation.

### INHIBITION OF HEPATIC MIXED-FUNCTION OXYGENASES BY CYTOTOXIC AGENTS *IN VITRO*.

A. Gescher and A. E. Green, *Cancer Chemotherapy Group, Department of Pharmacy, University of Aston, Birmingham.*

Inhibition of depression of drug-metabolizing enzymes may have severe clinical consequences in cancer chemotherapy, as many antitumour drugs exert their cytotoxic activity only after metabolic activation, predominantly in the liver. Most of them are also detoxified by the liver. If repeated treatment with a cytotoxic agent or its concurrent administration with other drugs as part of a combination regime implicates adverse effects on drug-metabolizing enzymes, altered bioactivation or detoxification patterns may cause diminished or changed therapeutic response to chemotherapy.

We investigated the ability of procarbazine, lomustine, carmustine, chlorozotocine, hexamethylmelamine, dacarbazine and the experimental antitumour triazene *p*-carbmethoxyphenyldimethyltriazene and some of their metabolites to inhibit the metabolic O-demethylation of p-nitroanisol in livers of male CBA LAC mice *in vitro*. This reaction is a model for metabolic functionalization reactions catalysed by mixed-function oxygenases (Netter, 1964, *J. Pharmacol. Exp. Ther.*, **146,** 61). Significantly, all the drugs studied require oxidative bioactivation. Dacarbazine and chlorozotocine showed no inhibition at all at concentrations < 5 mM. All the other agents interfered with drug-metabolizing enzyme activity. Dixon plots (Dixon, 1952, *Biochem. J.*, **55,** 170) indicated non-competitive or uncompetitive inhibition for most agents, with $K_i$ values in the $\mu$M or mM range. Procarbazine increased inhibition after 15 min preincubation in buffer before the addition of liver homogenate, which indicates that a metabolite of the labile drug molecule contributes to drug metabolism interactions with procarbazine.

### UNEXPECTED LOSS OF ANTI-TUMOUR ACTIVITY IN A HOMOLOGOUS SERIES OF 3-ALKYL-1-ARYL-3-METHYLTRIAZENES.

D. E. V. Wilman, P. J. Cox, P. M. Goddard and K. Merai, *Department of Biochemical Pharmacology, Institute of Cancer Research, London SW3.*

The continuing clinical interest in DTIC, despite its severe side effects, has prompted us to apply our earlier structure-activity results (Connors *et al.*, 1976, *Biochem. Pharmacol.*, **25,** 241) to this drug-design problem, in the search for a second-generation agent. An extended homologous series of 3-alkyl-1-(4-carboxyphenyl)-3-methyltriazenes shows unexpected loss of anti-tumour activity at alkyl chain lengths greater than 5 carbon atoms. Physicochemical parameters, in particular solubility at pH 7·4 and octanol-buffer partition coefficients, show no similar sudden breaks.

It has proved impossible to detect any *in vitro* dealkylation of these compounds, and we therefore turned our attention to the analogous 3 - alkyl - 1 -(4 - carbamoylphenyl) - 3 -methyl - triazenes, which exhibit a similar pattern of anti-tumour activity. In this series a considerable degree of *in vitro* demethylation and dealkylation occurs in compounds with anti-tumour activity, but both are markedly reduced in those lacking activity.

### *IN VITRO* CYTOTOXICITY OF HEXAMETHYLMELAMINE AND ITS ANALOGUES.

C. J. Rutty, G. Abel and K. R. Harrap. *Dept. of Biochemical Pharmacology, Institute of Cancer Research, London SW3.*

Hexamethylmelamine (HMM) and a number of its derivatives are toxic to PC6 plasmacytoma cells *in vitro*, and a number of N-methylmelamines have also shown significant activity against this tumour *in vivo*. N-methylolmelamines are significantly more toxic *in vitro* than HMM itself. These compounds break down to release formaldehyde, which is itself highly cytotoxic. Pentamethylmonomethylolmelamine rapidly inhibits the growth of PC6 cells in culture, whereas HMM and pentamethylmelamine (PMM) require prolonged contact with the cells in order to exert a cytotoxic effect. HMM and its metabolites are also toxic to a number of other cell lines *in vitro*, although without effect *in vivo*. The toxicity of N-methylols to L1210 and Walker ascites cells can be completely reversed using semicarbazide, whereas their toxicity to PC6 cells is irreversible, although in all cases the toxicity of formaldehyde itself can be reversed. These findings suggest that, in tumour cells which are sensitive *in vivo*, the N-methylols are able to exert a more selective effect than formaldehyde. HMM, PMM and PMM-methylol rapidly inhibit both DNA and RNA synthesis *in vitro*. These effects are rapidly and completely reversible for unmetabolized PMM after removal of the drug, but totally irreversible for the methylol, suggesting a different mechanism of action. The methylols may exert their effects on nucleic acid synthesis by direct inhibition of DNA template activity.

**EFFECT OF CHLORAMBUCIL ON NUCLEAR PROTEIN PHOSPHATASES.** P. J. Thraves, R. Wilkinson and K. R. Harrap, *Dept. of Biochem. Pharmacol., Institute of Cancer Research, Sutton, Surrey.*

The alkylating agent chlorambucil increases nuclear protein phosphorylation in Walker 256 ascites tumour cells sensitive to the toxic action of alkylating agents, but not in a paired line resistant to a wide range of alkylating agents. However, binary combinations of chlorambucil and the steroid prednisolone also increase nuclear protein phosphorylation in the resistant tumour. Fractionation of nuclear protein phosphatases and kinases show the phosphatases to be located in the nucleoplasm and the kinases in the non-histone chromosomal protein. 10-week-old female Wistar rats bearing either sensitive or resistant cells were treated with 10 or 20 mg/kg of chlorambucil. Tumour cells were collected 48 h later and nuclei prepared (Rickwood *et al.*, 1973, *Biochim. Biophys. Acta*, **299,** 162.). Phosphatase activity was determined by the decrease in activity of nuclei previously labelled by incubation with $\gamma^{32}$P-ATP. Neither chlorambucil nor a combination of chlorambucil plus prednisolone induces a significant inhibition of nuclear protein phosphatase activity in either alkylating-agent-sensitive, or resistant tumour cells. We cannot therefore explain the observed increase in total nuclear protein phosphorylation by a modification of phosphatase activity. Studies on the isolation and characterization of nuclear protein kinases are in progress to determine their role in the increased phosphorylation.

**FACTORS INFLUENCING THE PROTECTION OF TUMOUR CELLS FROM 5-FLUOROURACIL TOXICITY BY THYMIDINE.** J. F. R. Muindi, S. E. Barrie and K. R. Harrap, *Dept. of Biochemical Pharmacology, Institute of Cancer Research, Sutton, Surrey.*

There has been recent interest in the use of thymidine (TdR) to modulate the effects of 5-fluorouracil (FU) (Martin *et al.*, 1978 *Proc. Am. Ass. Cancer Res.*, **19,** 251). Based on the idea that TdR will supply the thymidylate the *de novo* synthesis of which is prevented by 5-fluorodeoxyuridylate, it should be possible to protect all cell lines from FU toxicity *in vitro*. This, however, is not the case (Reeves *et al.*, 1969, *Proc. Soc. Exp. Biol. Med.*, **131,** 1068). A study of the factors which affect the protection was undertaken to see whether there could be a rational basis for the use of FU/TdR combination in man. Ehrlich ascites tumour cells were protected from FU toxicity by TdR in tissue culture, whereas Walker carcinosarcoma 256 were not. Uridine failed to protect either of these cell lines. The incorporation of FU into RNA was not affected by TdR, and did not differ between the 2 cell lines. Studies of the enzyme responsible for TdR utilization (thymidine kinase) and degradation (dihydropyrimidine dehydrogenase DHPD) showed that the degradative pathway alone differed, being 24 pmol/h/mg protein in Walker carcinosarcoma 256 cells and undetectable in Ehrlich ascites tumour cells (*i.e.* < 8 pmol/h/mg protein). It is suggested that only those tissues with a low activity of DHPD will be protected from FU toxicity by TdR.

**2-AMINO-4-HYDROXY QUINAZOLINE ANALOGUES OF FOLIC ACID AS THYMIDYLATE SYNTHETASE INHIBITORS.** T. R. Jones, A. H. Calvert, A. L. Jackman, S. Brown and K. R. Harrap, *Dept. of Biochemical Pharmacology, Institute of Cancer Research, Sutton, Surrey.*

Certain quinazolines which mimic the folate rather than the pyrimidine substrate are effective inhibitors of thymidylate synthetase (TS) (Paine *et al.*, 1978, *Br. J. Cancer*, **38,** 180). Desirable features of these compounds are their independence of metabolic activation, and the improbability of their incorporation into nucleic acids which may be the cause of the delayed toxicity of FU. The substitution of a methyl group at the 10 position of H-(p(((2-amino-4-hydroxy-6-quinazolinyl)methyl)amino)benzoyl)–L-glutamic acid increases its ability to inhibit bacterial TS 10-fold (Bird *et al.*, 1970, *Mol. Pharmacol.*, **6,** 573). A series of these compounds, including other 10 substitutions, has been synthesized and evaluated, *viz*:

| House no. | 10 Substitution | $I_{50}$ for TS (nM) L1210 | $I_{50}$ for TS (nM) *L. casei* | $I_{50}$ for L1210 culture (μM) |
|---|---|---|---|---|
| CB 3705 | H | 570 | 860 | 2·2 |
| CB 3713 | Me | 42 | 64 | 4 |
| CB 3714 | Et | 27 | 38 | 16 |
| CB 3715 | Pr | 260 | 1100 | 40 |
| CB 3719 | Bu | 3200 | | 480 |

The inhibition of CB 3705 is competitive with 5,10-$CH_2$-THF ($K_i = 67$ nM, L1210 enzyme). Assuming that CB 3714 is also competitive, its $K_i$ will be 3·2 nM, which is comparable to 5FdUMP (Reyes & Heidelberger, 1965, *Mol. Pharmacol.*, **1,** 14). All of these compounds are reversible by thymidine in tissue culture. These studies may provide a "second generation" alternative to methotrexate (MTX) which will circumvent MTX resistance.

### PART II

### POSTERS

### TUMOUR INCIDENCE IN MICE AFTER OESTROGEN AND PROGESTERONE TREATMENT.

A. E. LEE, *Imperial Cancer Research Fund, London WC2.*

The widespread clinical use of ovarian hormones has led to renewed interest in the possible carcinogenicity of these compounds.

Oestrone (40 ng/ml drinking water) was given to 98 ovariectomized C57 BL mice continuously from 4–5 months of age until death, a mean period of 21·5 months (range 11–31). Half (Group 0 + P) received a total of 13 s.c. injections of progesterone (1·0 mg in oil) during weeks 1–4 and 26–39; at the same times the remainder (Group 0) received 0·5 ml vehicle.

At autopsy, no mammary, uterine or cervical tumours were found in either group. The most common abnormalities were lymphoid tumours or infiltrations. Most occurred in mice aged 24–30 months, in the following tissues (Group 0 given first); gut and mesenteries (46%, 46%); spleen (36%, 40%); liver (15% 21%); kidney (2%, 2%). Also, 4 subcutaneous lymphosarcomas were seen in Group 0. Haemangiomas were noticed in the liver (2%, 8%). Two mice in Group 0, and 1 in Group 0+P developed a spindle-cell sarcoma on the back (the site of injection).

When these results were compared with the data of Rowlatt *et al.* (1976, *Lab. Anim. Sci.*, **10,** 419), who used C57BL mice from the same breeding colony and kept under the same conditions, there were no significant differences in tumour distribution. It appears therefore that long-term administration of oestrogen, or oestrogen and progesterone, does not alter the normal organ distribution of carcinogenesis in ageing C57BL mice, nor induce tumours in target organs.

### THE EFFECT OF LYOPHILIZATION ON THE ACTIVITY OF OESTRADIOL AND PROGESTERONE RECEPTORS.

E. J. LLOYD, D. M. BARNES and L. G. SKINNER, *Clinical Research Laboratories, Christie Hospital and Holt Radium Institute, Manchester.*

The storage and transport of steroid hormone receptor-containing tissue is limited by the instability of the receptor proteins involved. Lyophilization of the tissue may yield a more stable product which would prove useful for quality-control investigations between laboratories.

The effect of lyophilization on the activity of the oestradiol ($ER_c$) and progesterone ($PR_c$) receptors in human breast-tumour tissue and normal uterine tissue, has been studied. $ER_c$ and $PR_c$ activity in cytosol was determined by a dextran-charcoal (DCC) method (Korenman & Dukes, 1970, *J. Clin. Endocrinol.* **30,** 659) using $^3$H-oestradiol and $^3$H-R5020 respectively. Oestrogen receptor activity in the "nuclear" debris ($ER_n$) was determined by the method of Laing *et al.*, (1977, *Lancet*, ii, 168). The dissociation constant ($K_D$) and number of receptor sites were calculated from Scatchard analyses of the data.

From the results obtained the activity in the lyophilized tissue was expressed as a percentage of the activity prior to lyophilization. The mean values for $ER_c$ (17 samples) $PR_c$ (5 samples) and $ER_n$ (8 samples) were 94%, 112%, and 157% respectively. The dissociation constants did not differ significantly.

Receptor activity, therefore, survives lyophilization for both $ER_c$ and $PR_c$ and lyophilized tissue can subsequently be stored without loss of activity. This procedure may be advantageous in receptor purification, and at present lyophilized tissue is being used for quality control.

### GLYCOPROTEIN HORMONE α SUBUNIT AND SURVIVAL IN MELANOMA PATIENTS.

I. A. MACFARLANE, N. THATCHER*, R. SWINDELL, C. G. BEARDWELL, E. HAYWARD and D. CROWTHER*, *Endocrinology Department and *CRC Department of Medical Oncology, Christie Hospital and Holt Radium Institute, Manchester.*

The common α subunit of the glycoprotein hormones (LH, FSH, TSH and HCG) is raised in the blood of patients with various malignant conditions (Dosogne-Guerin *et al.*, 1978; *Eur. J. Cancer*, **14,** 525). We measured serum α subunit levels in 55 patients with melanoma before and during chemoimmunotherapy. The upper limit of normal in men and premenopausal women was 2·3 ng/ml and in postmenopausal women 9·5 ng/ml.

In 20 premenopausal patients $\alpha$ levels before therapy ranged from $<0{\cdot}5$ to 10 ng/ml, median 2·3 ng/ml. 10 had levels above the upper limit of normal, median 6·8 ng/ml. The median survival in patients with elevated $\alpha$ levels was 19 weeks, compared to a median survival of 83 weeks in those with normal $\alpha$ levels. This difference was significant ($P=0{\cdot}015$, Log rank).

In 11 postmenopausal patients $\alpha$ levels before therapy ranged from 0·5 to 13 ng/ml, median 3·5 ng/ml. Median survival of these 11 patients was 26 weeks. Only 1 had a raised $\alpha$ level, and this patient died at 20 weeks. In 24 male patients, $\alpha$ levels before therapy ranged from $<0{\cdot}5$ to 10 ng/ml, median 0·5 ng/ml. The median survival of these 24 patients was 32 weeks. Only one had an elevated $\alpha$ level, and this patient died at 8 weeks.

During therapy, 1 further premenopausal, 1 postmenopausal and 4 male patients developed $\alpha$ levels above the upper limit of normal. Maximum $\alpha$ levels during treatment, levels at documented regression or progression of disease and levels at the end of therapy, did not significantly influence survival of patient groups.

Conclusions: $\alpha$ subunit levels are raised in up to 50% of premenopausal patients with melanoma before treatment and are associated with reduced survival. In men and postmenopausal women $\alpha$ levels are infrequently raised.

**COMPARISON OF PAM AND CEA AS DETECTORS OF MAMMARY MICROMETASTASES.** J. MAXWELL ANDERSON*, S. K. JHUNJHUNWALA*, J. M. EWAN*, W. H. STIMSON† and G. GETTINBY†, **Royal Infirmary, Glasgow and †University of Strathclyde, Glasgow.*

Nineteen of 61 Stage I or II mammary-cancer bearers developed conventionally detectable metastases within 8–44 (median 15) months of commencing follow-up. Serum pregnancy-associated macroglobulin (PAM, PAG, $\alpha_2$ PAG) rose by $>75\%$ above baseline values before detection of metastases in all but 4 of these 19 patients and in 11/42 remaining well. The means of the maximum percentage rises in PAM were 286 for the metastatic and 67 for the well patients ($P<0{\cdot}01$ in a Mann-Whitney U-test). In contrast there was a similar rise of serum carcinoembryonic antigen (CEA) in only 6/19 patients with metastases (1 other rose $>75\%$ above the baseline but fell before metastases appeared) and 13/42 clinically well patients had rises greater than the 75% level. The predictive performance of CEA was not bettered by taking a 45% rise as the discriminant, or by using a function of the combined rises in PAM and CEA, and the difference between the maximum percentage rises in the metastatic (92) and the well (106) patients was not significant. Thus CEA changes do not discriminate between those about to manifest macrometastases and those remaining well, but PAM rises exceeding 75% often do this with apparent odds of nearly 2 to 1 on the clinical appearance of these metastases within a year or so.

**MORPHOLOGICAL FEATURES OF THIRTEEN MURINE MAMMARY TUMOUR CELL LINES.** A. J. COCHRAN, N. KUZUMAKI, I. A. R. MORE and G. KLEIN, *Pathology Department, Western Infirmary, Glasgow, and Dept. Tumour Biology, Karolinska Institute, Stockholm, Sweden.*

Seventeen transplantable spontaneous and virus-induced mammary tumours from various strains of mice were cultured in an effort to obtain established cell lines. This was successfully achieved with 13 lines, which on regular subculture grew as monolayers and produced tumours in syngeneic mice. Histological examination of such tumours showed that 7 were adenocarcinomas, 3 were solid carcinomas and 3 had a spindle-cell morphology associated with abundant pericellular reticulin and collagen. The light-microscopic appearances of the 3 spindle-celled tumours suggested that they were sarcomas, but ultrastructural examination showed features characteristic of epithelial cells, proving them to be spindle-celled carcinomas.

**SURFACE FEATURES OF TRANSPLANTABLE MOUSE COLON CANCERS.** J. A. DOUBLE*, M. C. BIBBY* and J. C. KNOWLES†, **Postgraduate School of Studies in Medical and Surgical Sciences, University of Bradford, and †Unit for Cancer Research, School of Medicine, University of Leeds.*

We have previously reported the development and characterization of an ascitic line from a solid transplantable mouse adenocarcinoma of the colon. (Double & Cifuentes de Castro, 1978 *Cancer Treat. Rep.*, **62**, 85). Using the methods described we have produced another ascitic variant designated MAC 13/A from the solid line MAC 13. The histology of MAC 13 has been previously described (Double *et al*, 1975, *J. Natl Cancer Inst.*, **54**, 27). This study describes the surface features of the cells in the solid ascitic lines.

Initially the tumour fragments consist of individual cells attached by desmosomes. Free surfaces of the cells adjacent to lumina bear microvillus-like processes. Tumour cells adjacent to the lumen are attached by typical epithelial junctional complexes comprising zonula occludens, zonula adherens and desmosomes. The ascites line consists of either single cells or clumps. Scanning electron microscopy indicated irregular folding of the cell surfaces. Transmission electron microscopy revealed that the clumps of cells consist of individual tumour cells attached by desmosomes and tight junctions. Microvillus-like structures project from the free surfaces of the cells into intercellular lumina or vesicles. The considerable degree of differentiation shown by these clumps of cells would emphasize the usefulness of these tumour lines in experimental chemotherapy.

Future studies will examine the ultrastructure of systemic tumours produced by i.v. inoculation of ascites cells.

**ESTABLISHMENT OF LONG-TERM CELL CULTURES FROM EQUINE SARCOIDS.** A. DOYLE, L. N. OWEN and T. D. LITTLEWOOD, *Department of Clinical Veterinary Medicine, University of Cambridge*

Equine sarcoids are spontaneous connective-tissue tumours which can occur at many different sites. Histologically they appear as fibromas or low-grade fibrosarcomas, and have a high tendency to recur after excision. They have some pathological resemblance to the human dermatofibrosarcoma protuberans. Evidence of cell-free transmission is not totally convincing, but the tumours may have a viral origin, and this is being investigated.

In the establishment of tissue cultures the first technique used for the disaggregation of biopsy material involved incubating minced tissue in 0·25% trypsin/Hanks' BSS with antibiotics at 37°C for 3–4 and 16–20 h. This led to the successful culture (*i.e.* $>20$ subcultures) of 2/7 tumour samples; both were derived by long-term trypsinization. However, this has been superseded by a more successful technique in which the minced tumour tissue is incubated at 37°C in normal growth medium supplemented with antibiotics, and containing 200 u/ml collagenase. A well-dispersed cell suspension is given after incubation for 24–72 h. Thus 16/25 tumours have been established in culture. The added advantage of this method is that tissue can be transported in the disaggregation medium, so enabling a larger number of samples to be obtained from clinicians in distant parts of the country. Cultures have been established from tumour material received up to 6 days after excision; the only major problem which remains is gross contamination from the tumours which often show secondary infection.

The research is supported by the Horserace Betting Levy Board.

**INHIBITION OF MEMBRANE ATP-ASE ENZYMES BY ANTITUMOUR ALKYLATING AGENTS.** J. A. HICKMAN and G. E. SPURGIN, *Cancer Chemotherapy Group, Department of Pharmacy, University of Aston, Birmingham, B4.*

It has been suggested that alkylation of DNA may not alone be able to account for the anti-tumour activity of alkylating agents (Wheeler, 1967, *Fed. Proc.*, **26,** 885). We have considered plasma membrane ATPase enzymes as possible targets for the alkylating agents which may explain their cytotoxicity. These enzymes are implicated in the control of cell proliferation (Quastel & Kaplan, 1970, *Exp. Cell. Res.*, **62,** 407; Sanui & Rubin, 1978, *J. Cell Physiol.*, **96,** 265) have raised activity in transformed cells (Kasorov & Friedman, 1974, *Cancer Res.*, **34,** 1862) and are inhibited by alkylating agents (Skou, 1963, *Biochem. Biophys. Res. Commun.*, **10,** 79).

A study has been made of the enzymes from a plasma-cell tumour (PC6) which is sensitive to alkylating agents *in vivo*, and we have characterized $Na^+K^+$ ATPase (E.C.3.6.1.3)$Mg^{2+}$ ATPase, an external $Ca^{2+}$ ATPase and *p*-nitrophenolphosphatase (E.C.3.1.3.1) activities in a crude cell-membrane preparation from this tumour. Nitrogen mustard (HN2) inhibits in a time-dependent manner 90% of $Na^+K^+$ ATPase activity at $9\times10^{-15}$M, $Mg^{2+}$ ATPase ($ID_{90}=7\times10^{-15}$M) and has little effect on either external $Ca^{2+}$ ATPase ($ID_{90}>10^{-4}$M) or *p*-nitrophenylphosphatase ($ID_{90}>10^{-4}$M). A comparison of nitrogen mustard with monofunctional alkylating agents (*e.g.* iodoacetate, N-ethylmaleimide) showed it to be by many orders of magnitude more potent as an inhibitor of $Na^+K^+$ATPase.

**CYTOTOXIC–NON-CYTOTOXIC DRUG INTERACTION.** J. F. B. STUART*, and I. H. STOCKLEY†, **Department of Clinical Oncology, University of Glasgow, and* †*Department of Pharmaceutical Chemistry, Nottingham University.*

As a group, patients on cytotoxic chemotherapy are at the top of the bad-risk league with regard to possible drug interactions. The drug-interaction alert chart (Stockley), Medidisc (Whiting) and the booklets, Safer Prescribing (Beeley) and Drug Interaction Guide (Abbot) are of help to the physician. However these publications fall short in only providing limited information relating to cytotoxic–non-cytotoxic drug interactions. A chart has been prepared to illustrate not only the possible cytotoxic–non-cytotoxic adverse interactions, but also some of those interactions which occur between cytotoxic drugs themselves. An additional feature of the chart is that it highlights the non-cytotoxic–cytotoxic adverse interactions pertaining to that group of drugs on the chart. The specialized nature of oncology and the need to emphasize the clinical significance of possible adverse interactions are met by a simple reference card system to be used in conjunction with the chart.

**CHROMOGENIC SUBSTRATE ASSAY FOR PLASMINOGEN ACTIVATOR, USING LEWIS LUNG CARCINOMA AND SV40-3T3 CELLS IN TISSUE CULTURE.** P. WHUR, J. BOSTON, M. MAGUDIA and D. C. WILLIAMS, *Cell Biology Unit, Marie Curie Memorial Foundation, The Chart, Oxted, Surrey.*

A simple and rapid liquid assay for plasminogen activator (PA) production by cells in tissue culture has been developed, which is nontoxic and uses only commercially available products.

Cells in 35 mm Petri dishes were incubated for 4 h in 0·8 ml serum-free indicator-free medium (Flow Laboratories) containing plasminogen (KabiVitrum, 0·5 cu/ml) and the chromogenic substrate S-2251 (KabiVitrum, 385 $\mu$g/ml). Activation converts the plasminogen to plasmin, which degrades S-2251 to release a yellow dye. Changes in optical density ($OD_{405}$) were calibrated against a plasmin (KabiVitrum, $\leq$0·1 cu/ml) standard curve, to measure the PA activity of the cells.

The assay was extended to 24 h by adding 10% serum free of plasminogen and inhibitors of plasmin (Northumbria Biologicals). High backgrounds were generated by the serum, but this form of the assay allowed longer-term studies in growth-supporting media.

Changes in $OD_{405}$ were linear with activator concentration when urokinase (Leo Laboratories, $\leq$5 cu/ml) was used. The plasmin standard curve was also linear, but small amounts of plasmin were lost on the vessel walls. PA production was linearly related to cell density in the 2 cases examined. The preferred number of cells ranged from a few thousand to a million, depending upon their PA activity. Cells which generated high levels of plasmin rounded up slightly owing to proteolysis, but they were otherwise unaffected by the assay and could be re-used.

**AN EPIGENETIC MODEL FOR THE GENESIS OF PARANEOPLASTIC SYNDROMES.** G. V. SHERBET, *Cancer Research Unit, Royal Victoria Infirmary, Newcastle upon Tyne.*

Cell differentiation is known to involve a progressive determination and restriction of competences and a sequential gene activation. It is proposed that neoplastic changes include a process of dedetermination and an acquisition of a previous state of competence, and that this leads to an abnormal activation of genes.

Paraneoplastic syndromes of endocrine dysfunction can be classified on an epigenetic basis of the degree of dedetermination which the neoplasms may have undergone. It is proposed that Group I neoplasms have acquired the preceding competence (Group IA, *e.g.* bronchial carcinoma—syndrome hyperadrenocorticism; both neoplasm and endocrine syndrome of bronchial endoderm origin), or one of several earlier stages of competence (Group IB, *e.g.* primary hepatoma—syndrome hypercalcaemia, both endodermal in origin but not as closely related as in IA). Group II tumours (*e.g.* renal carcinoma—syndrome erythrocytosis) have to undergo more extensive dedetermination than Group I. Group III neoplasms (*e.g.* adrenocortical tumour, mesodermal origin—syndrome hypoglycaemia, endoderm-derived endocrine function) show a highly labile state of determination. This epigenetic classification seems to correlate with the pattern of frequency of incidence of paraneoplastic syndromes (Sherbet, 1974, *Ann. N.Y. Acad. Sci.*, **230,** 524).

**DEMONSTRATION OF HUMAN AND MOUSE COMPONENTS OF HETERO-TRANSPLANTED TUMOURS.** H. M. WARENIUS and N. M. BLEEHEN, *MRC Clinical Oncology Unit, Hills Road, Cambridge.*

It has been demonstrated that, with the exception of foetal tissues (Povlsen *et al.*, 1974, *Nature*, **248,** 247) normal cells will not proliferate when injected into immunosuppressed mice (Stanbridge, *et al.*, 1975, *Cancer Res.*, **35,** 2203. It might thus be expected that human tumours transplanted to immunosuppressed mice would acquire a mouse stroma. This possibility has been investigated using fluorescein-conjugated IgG fractions prepared from the sera of rabbits immunized with either pooled human buffy-coat cells or mouse thymocytes. Paraformaldehyde-fixed frozen sections were stained with these immunoglobulins and counterstained with propidium iodide. Antibody specificity was checked on frozen sections of mouse and human colon and on a human lung squamous-cell carcinoma. Application of the technique to a human colonic adenocarcinoma cell line, 2 squamous-cell lung carcinomas and a mucin-secreting lung adenocarcinoma grown as xenografts in immunosuppressed mice showed that, whilst the main tumour mass stained with anti-human serum but not with anti-mouse serum, the stromal and vascular elements stained distinctively with the anti-mouse serum but not the anti-human serum. Differential counting of photographs of stained frozen sections revealed about 75% of the tumour mass to be of human origin and about 25% to be mouse.

These studies thus confirm the chimaeric nature of human tumours heterotransplanted to immunosuppressed mice. Results obtained with this model system may thus need to be interpreted in the light of these findings.